# Parkin regulates neuronal lipid homeostasis through SREBP2-lipoprotein lipase pathway—implications for Parkinson’s disease

**DOI:** 10.1093/hmg/ddac297

**Published:** 2022-11-26

**Authors:** Willcyn Tang, John Thundyil, Grace Gui Yin Lim, Teddy J W Tng, Sean Qing Zhang Yeow, Aditya Nair, Chou Chai, Tso-Pang Yao, Kah-Leong Lim

**Affiliations:** Department of Research, Lee Kong Chian School of Medicine, Nanyang Technological University, Singapore 308232, Singapore; Department of Research, National Neuroscience Institute, Singapore 308433, Singapore; Department of Research, National Neuroscience Institute, Singapore 308433, Singapore; Department of Research, National Neuroscience Institute, Singapore 308433, Singapore; Department of Research, Lee Kong Chian School of Medicine, Nanyang Technological University, Singapore 308232, Singapore; Graduate School of Integrative Sciences and Engineering, National University of Singapore, Singapore 119077, Singapore; Department of Research, National Neuroscience Institute, Singapore 308433, Singapore; Department of Research, Lee Kong Chian School of Medicine, Nanyang Technological University, Singapore 308232, Singapore; Department of Pharmacology and Cancer Biology, Duke School of Medicine, Duke University, Durham, NC 27710, USA; Department of Research, Lee Kong Chian School of Medicine, Nanyang Technological University, Singapore 308232, Singapore; Department of Research, National Neuroscience Institute, Singapore 308433, Singapore; Department of Brain Sciences, Imperial College London, London SW7 2AZ, UK

## Abstract

Abnormal lipid homeostasis has been observed in the brain of Parkinson’s disease (PD) patients and experimental models, although the mechanism underlying this phenomenon is unclear. Notably, previous studies have reported that the PD-linked protein Parkin functionally interacts with important lipid regulators, including Sterol Regulatory Element-Binding Proteins (SREBPs) and cluster of differentiation 36 (CD36). Here, we demonstrate a functional relationship between Parkin and lipoprotein lipase (LPL), a triglyceride lipase that is widely expressed in the brain. Using a human neuroblastoma cell line and a Parkin knockout mouse model, we demonstrate that Parkin expression level positively correlates with neuronal LPL protein level and activity. Importantly, our study identified SREBP2, a major regulator of sterol and fatty acid synthesis, as a potential mediator between Parkin and LPL. Supporting this, SREBP2 genetic ablation abolished Parkin effect on LPL expression. We further demonstrate that Parkin-LPL pathway regulates the formation of intracellular lipid droplets, and that this pathway is upregulated upon exposure to PD-linked oxidative stress induced by rotenone. Finally, we show that inhibition of either LPL or SREBP2 exacerbates rotenone-induced cell death. Taken together, our findings reveal a novel pathway linking Parkin, SREBP2 and LPL in neuronal lipid homeostasis that may be relevant to the pathogenesis of PD.

## Introduction

Parkinson’s disease (PD) is a prevalent neurodegenerative disease affecting >6 million individuals globally (1). Clinically, the disease is attended by a constellation of motoric deficits that progressively worsen with age, which ultimately leads to near total immobility. Although pathological changes are distributed in the PD brain (2), the principal lesion that underlies the characteristic motor phenotype of PD patients is unequivocally the loss of dopaminergic (DA) neurons in the substantia nigra pars compacta (SNpc) of the midbrain. As DA neurons are known to be highly energy-demanding and vulnerable to metabolic insults (3,4), metabolic dysregulation has been proposed as an early pathological event that precedes neurodegeneration in PD. Interestingly, several genetic risk factors for PD (e.g. *SNCA*, *GBA* and *SREBF1*) are associated with lipid homeostasis, suggesting a potential involvement of lipid dysregulation in PD pathogenesis (5–8).Consistent with this notion, several studies have reported abnormal lipid metabolism in brain tissues from PD patients and various experimental models of PD. For example, Halliday *et al.* (9) reported an increase in the density of lipid-containing neuromelanin organelles in the surviving SNpc DA neurons from the brains of patients with early stage PD compared to those of healthy controls. At the cellular level, α-Synuclein-enriched Lewy bodies (LBs), the pathological hallmark of PD, are decorated with lipid-enriched, densely packed vesicular structures and dysmorphic organelles (10–12). Furthermore, α-Synuclein overexpression (O/E) or cellular exposure to PD-relevant toxins promotes the accumulation of intracellular lipid droplets (LDs) and subsequent cell death (13–15). Notably, LDs are known to interact with and regulate the function of mitochondria, the dysfunction of which is also linked to PD pathogenesis (16–18). Notwithstanding the prominent relationship between altered lipid homeostasis and PD, the underlying molecular mechanism remains not well clarified. Interestingly, Kim *et al.* (19) reported about a decade ago that Parkin, a ubiquitin ligase whose mutations cause recessive early-onset Parkinsonism, regulates lipid uptake in non-neuronal cells by stabilizing cluster of differentiation 36 (CD36), which is involved in transporting fatty acid (FA), via mono-ubiquitination. In a more recent development, Ivatt *et al.* (7) reported that Parkin functionally interacts with Sterol Regulatory Element-Binding Protein −1 (SREBP1) and −2 (SREBP2), which are major regulators of lipid metabolism, but in the context of mitophagy. Prompted by these observations, we were interested to pursue the role of Parkin in lipid homeostasis.

In the present study, we demonstrated by means of multiple experimental models that Parkin regulates the SREBP2-lipoprotein lipase (LPL) pathway. We found that Parkin expression is positively correlated with the levels of LPL and its upstream regulator SREBP2. Moreover, CRISPR-mediated ablation of *SREBP2* abolished this relationship, which links the three components in a shared pathway. Alongside this, we showed that Parkin downregulates F-box And WD Repeat Domain Containing 7 (FBXW7)—a known component of SCF^Slimb^ E3 ubiquitin ligase complex that targets nuclear SREBPs for degradation (20). We further showed that Parkin-LPL pathway regulates the intracellular deposition of LDs. Interestingly, the Parkin-SREBP2-LPL pathway is upregulated upon cellular exposure to PD-linked oxidative stress (i.e. rotenone), which is known to elevate LD production. Inhibition of either LPL or SREBP2 disrupts this phenomenon and exacerbates rotenone-induced cell death. Taken together, our findings reveal a novel pathway implicating Parkin, SREBP2 and LPL in neuronal lipid homeostasis and LD formation in a PD-relevant context.

## Results

### Parkin regulates LPL expression and activity

**Figure 1:**
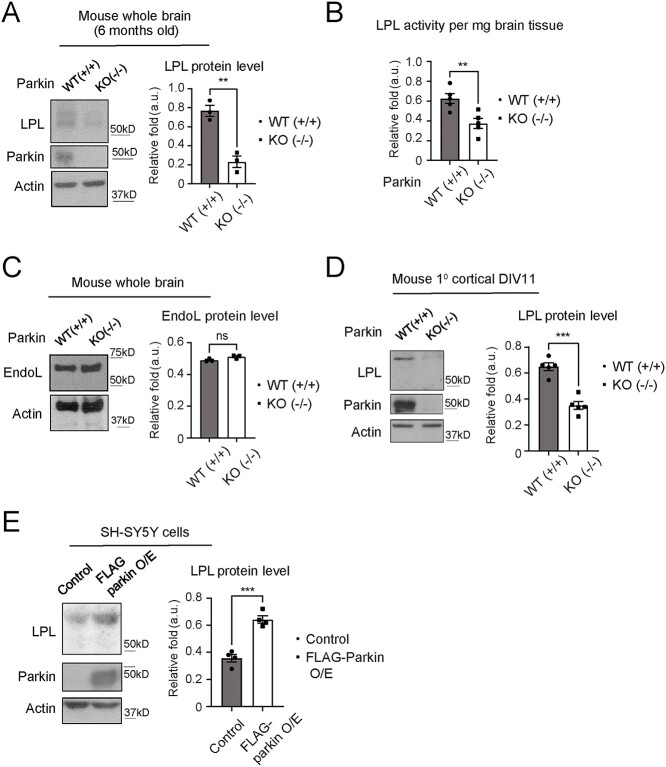
Parkin promotes LPL expression in neurons. (**A**) LPL protein expression level (detected with LPL 5D2 antibody) in whole brain lysates from 6 months old Parkin (+/+) and Parkin mice (−/−) (^*^^*^P < 0.01 (*t*-test), *n* = 3 animals each genotype). (**B**) LPL hydrolysis activity measurements in whole brain tissues from adult (3–6 months old) Parkin (+/+) and Parkin mice (−/−) (^*^^*^P < 0.01 (*t*-test), *n* = 5 animals each genotype). (**C**) EndoL protein expression level in whole brain lysates from Parkin (+/+) and Parkin mice (−/−) (n.s. = not statistically significant, *n* = 3 animals each genotype) (**D**) LPL protein expression level (detected with LPL A.4 antibody) in mouse primary cortical neuron lysates (DIV11) derived from Parkin (+/+) and Parkin (−/−) E17-18 embryos (^*^^*^^*^P < 0.001 (*t*-test), *n* = 5 biological replicates). (**E**) LPL protein expression level in SH-SY5Y cell line stably overexpressing FLAG-Parkin compared to cells with vector controls (^*^^*^^*^P < 0.001 (*t*-test), *n* = 4 biological replicates). Actin serves as protein loading controls for (A–E).

Given the report that Parkin modulates FA uptake in non-neuronal cells via stabilizing the expression of CD36 [a FA translocase; (19)], we were interested to examine if Parkin also exhibits a similar function in the brain. To address this, we measured the expression level of CD36 in the brains of mouse with homozygous deletion of exon 7 of *PARK2* gene [herein referred to as Parkin knockout (KO) mouse; (21)] and their wild-type (WT) counterparts. In contrast to the findings by Kim *et al*., we found that brain CD36 protein expression level was not affected by the genetic ablation of Parkin (Supplementary Material, Fig. S1A). Given the close functional relationship of LPL and CD36 in the regulation of FA uptake (22), we then measured LPL protein expression in Parkin WT and KO mouse brains. Interestingly, loss of Parkin results in a dramatic reduction of LPL protein amount in the Parkin KO mouse brains compared with Parkin WT brains (Fig. 1A and Supplementary Material, Fig. S14). Moreover, LPL lipid hydrolysis assay revealed a significant decrease in LPL activity in Parkin KO mouse brain lysates compared with those of control mice (Fig. 1B). To ascertain the specificity of Parkin effect on LPL, we measured the protein expression of a related lipase, namely Endothelial Lipase (EndoL), but found no apparent difference in its expression between Parkin WT and KO mouse brain lysates (Fig. 1C and Supplementary Material, Fig. S14). Notably, we also detected significant reductions of LPL level in the heart, muscle and liver tissues from Parkin KO mice compared with their control counterparts (Supplementary Material, Fig. S1B–D). Further experiments with primary mouse cortical neurons also showed that LPL protein expression level is markedly reduced in Parkin KO neurons (Fig. 1D and Supplementary Material, Fig. S14).

To further investigate the effect of Parkin on LPL level, we employed SH-SY5Y neuroblastoma cell model with stable O/E of FLAG-tagged Parkin that we have previously reported (23). Immunoblotting revealed that LPL expression level is significantly elevated in Parkin O/E cells compared with control cells (Fig. 1E and Supplementary Material, Fig. S14). However, our co-immunoprecipitation experimental failed to detect any appreciable physical interaction between Parkin and LPL in SH-SY5Y cells co-expressing FLAG-Parkin and Myc-LPL (Supplementary Material, Fig. S2A and B). Immunoprecipitated Parkin was able to pull down Hsp70, a previously reported interactor (24). Taken together, our data suggest that Parkin positively regulates LPL expression level in neuronal cells and that this functional interaction is likely to involve other player(s).

### Modulation of LPL level by Parkin is SREBP2-dependent

**Figure 2:**
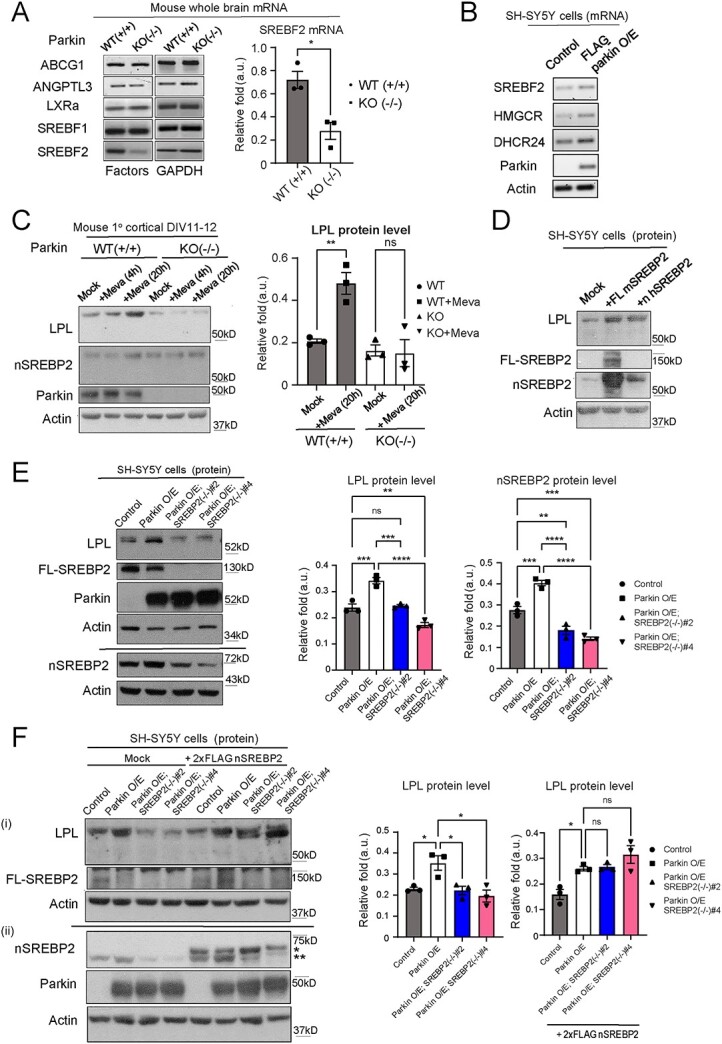
Parkin regulates LPL expression through SREBP2. (**A**) RT-PCR measurements of mRNA levels of genes known as positive or negative regulator of LPL expression in whole brain lysates from adult Parkin (+/+) and (−/−) mice (^*^  *P* < 0.05 (*t*-test), *n* = 3 animals each genotype) (*ABCG1* = ATP Binding Cassette Subfamily G Member 1; *ANGPTL3* = Angiopoietin-like protein 3; *LXRa* = Liver X Receptor Alpha; *SREBF1* & *-2* = Sterol Regulatory Element-Binding Transcription Factor 1 & 2). *GAPDH* mRNA level serves as loading controls. For other quantification see Supplementary Material, Fig. S3A (**B**) RT-PCR measurements of mRNA levels of *SREBF2* and its downstream transcription targets, *HMCGR* and *DHCR24*, in lysates from SH-SY5Y cells stably overexpressing FLAG-Parkin or vector control (*HMGCR* = 3-Hydroxy-3-Methylglutaryl-CoA Reductase; *DHCR24* = 24-Dehydrocholesterol Reductase). Actin mRNA level serves as loading controls. For quantification see Supplementary Material, Fig. S3B (**C**) LPL protein expression levels (detected with LPL A.4 antibody) in Parkin (+/+) and Parkin (−/−) mouse primary cortical neurons DIV12-14 treated with the HMCGR inhibitor, Mevastatin (20 μM) for either 4 or 20 h compared with mock-treated cells (n.s. = not statistically significant; ^*^^*^P < 0.01 (ANOVA), *n* = 3 biological replicates for 20 h treatment). (**D**) LPL protein expression level (detected with LPL A.4 antibody) in SH-SY5Y cells exogenously expressing either 2xFLAG human nSREBP2 or mouse FL-SREBP2 compared with mock-treated cells. (**E**) Immunoblot showing LPL protein expression levels (detected with LPL A.4 antibody) in SREBF2 Δex3-15; FLAG-Parkin O/E SH-SY5Y cells compared with control cells (n.s. = not statistically significant; ^*^^*^P < 0.01; ^*^^*^^*^P < 0.001; ^*^^*^^*^^*^P < 0.0001 (ANOVA), *n* = 3 biological replicates). Loss of FL-SREBP2 expression and knockdown of nSREBP2 in Parkin O/E; SREBP2(−/−)#2 and -#4 cells are also shown. (**F**) (i) LPL (detected with LPL A.4 antibody), FL-SREBP2, and (ii) nSREBP2 protein expression levels in FLAG-Parkin O/E; SREBP2(−/−) and control SH-SY5Y cells with or without exogenous expression of 2xFLAG human nSREBP2. (^*^ protein band is 2xFLAG nSREBP2; ^*^^*^ protein band is endogenous nSREBP2). Figure (i) and (ii) are from identical samples. Actin serves as protein loading controls (n.s. = not statistically significant; ^*^P < 0.05, (ANOVA), *n* = 3 biological replicates).

LPL is known to be positively or negatively regulated by several molecules at the transcript or protein level (25). Therefore, we subsequently measured mRNA levels of known regulators of LPL in brain samples obtained from Parkin WT and KO mice. From this candidate screening, we found that the transcript level of sterol regulatory binding factor-2 (*SREBF2*) is significantly downregulated in Parkin KO brain lysates compared with those of control brains (Fig. 2A and Supplementary Material, Fig. S3A). *SREBF2* gene encodes SREBP2 protein, a key transcription factor for lipid and cholesterol biosynthesis. Notably, SREBP2 has been reported to positively regulate the expression level of LPL in preadipocyte cells (26). To further determine if Parkin affects SREBP2 activity, we quantified the mRNA levels of *SREBF2* and known SREBP2 transcriptional targets, namely *HMGCR* and *DHCR24*, in SH-SY5Y cells stably O/E Parkin via RT-PCR (Fig. 2B and Supplementary Material, Fig. S3B). Our results revealed that increase in Parkin expression significantly elevates the mRNA levels of these genes, suggesting that Parkin promotes SREBP2 downstream signaling.

Our observations suggest the possibility of Parkin promoting LPL expression via upregulating SREBP2 signaling. However, SREBP2 may have differential transcriptional targets in neurons (27). To test if SREBP2-LPL axis exists in neuronal cells, we treated primary mouse cortical neurons with the HMGCR inhibitor, Mevastatin, which activates SREBP2 signaling by promoting cholesterol depletion (28). Parkin WT primary neurons treated with Mevastatin (20 μM) for 20 h exhibit enhanced levels of both nuclear SREBP2 (nSREBP2, active form, molecular weight, MW = ~ 70 kDa) and LPL (Fig. 2C and Supplementary Material, Fig. S3C and Fig. S15). Interestingly, Mevastatin effects on nSREBP2 and LPL levels were abolished in Parkin KO neurons, indicating that Parkin is required for SREBP2-dependent LPL upregulation in neurons (Fig. 2C and Supplementary Material, Fig. S3C). To further corroborate this finding, we exogenously expressed human nSREBP2 (29) or mouse full-length SREBP2 (FL-SREBP2, MW = ~ 130 kDa) (30) through its respective DNA construct in SH-SY5Y cells. Transient expression of each construct increases LPL protein levels in these cells (Fig. 2D and Supplementary Material, Fig. S15). Thus, increase in nSREBP2 alone is sufficient to promote neuronal LPL expression.

To validate if Parkin modulates LPL level through SREBP2 signaling, we generated SREBP2 (−/−) KO cells via CRISPR-Cas9 method (31). Cas9 enzyme and two guide RNAs (gRNAS) targeting exon 3 and 15 of human SREBP2 gene were co-expressed in SH-SY5Y cells with Parkin O/E to induce a large genomic deletion of the SREBP2 gene (Δ exon 3–15; Supplementary Material, Fig. S4). Independent stable SREBP2 KO; Parkin O/E clones [i.e. P2–P5 (−/−)] were subsequently selected by means of their resistance to Puromycin. RT-PCR analysis demonstrated that these clones do not express SREBF2 transcript, but still retain their Parkin O/E phenotype (Supplementary Material, Fig. S4). Consistent with this, immunoblotting revealed the loss of FL-SREBP2 protein expression in Parkin O/E; SREBP2(-/-)#2 and -#4 (i.e. P2(−/−) and P4(−/−)) clones (Fig. 2E and Supplementary Material, Fig. S15). Curiously, we detected faint protein bands corresponding to nSREBP2, which may refer either the existence of unspecific background band at the similar MW of nSREBP2 or incomplete nSREBP2 KO. In the unlikely event that the latter scenario is true, the expression of nSREBP2 protein levels in this case is clearly significantly reduced in P2(−/−) and P4(−/−) clones compared with those of control and Parkin O/E parental cells (Fig. 2E), and is expected to affect the Parkin-SREBP2-LPL axis. Importantly, we found that CRISPR/Cas9-mediated KO of FL-SREBP2 abolishes the effect of Parkin O/E on LPL levels in these edited cells (Fig. 2E). Since CRISPR-Cas9 method may cause unwanted off-target effects (32), we performed a genetic rescue experiment by exogenously expressing nSREBP2 in the SREBP2 KO clones. Reintroduction of nSREBP2 into SREBP2 KO; Parkin O/E clones restored LPL protein expression to a comparable level to that of Parkin O/E cells (Fig. 2F and Supplementary Material, Fig. S15). Taken together, our results demonstrate that Parkin-mediated increase in LPL expression requires SREBP2, suggesting that Parkin-SREBP2-LPL are all linked in the same pathway.

### Parkin regulates nSREBP2 level by downregulating FBXW7β

**Figure 3:**
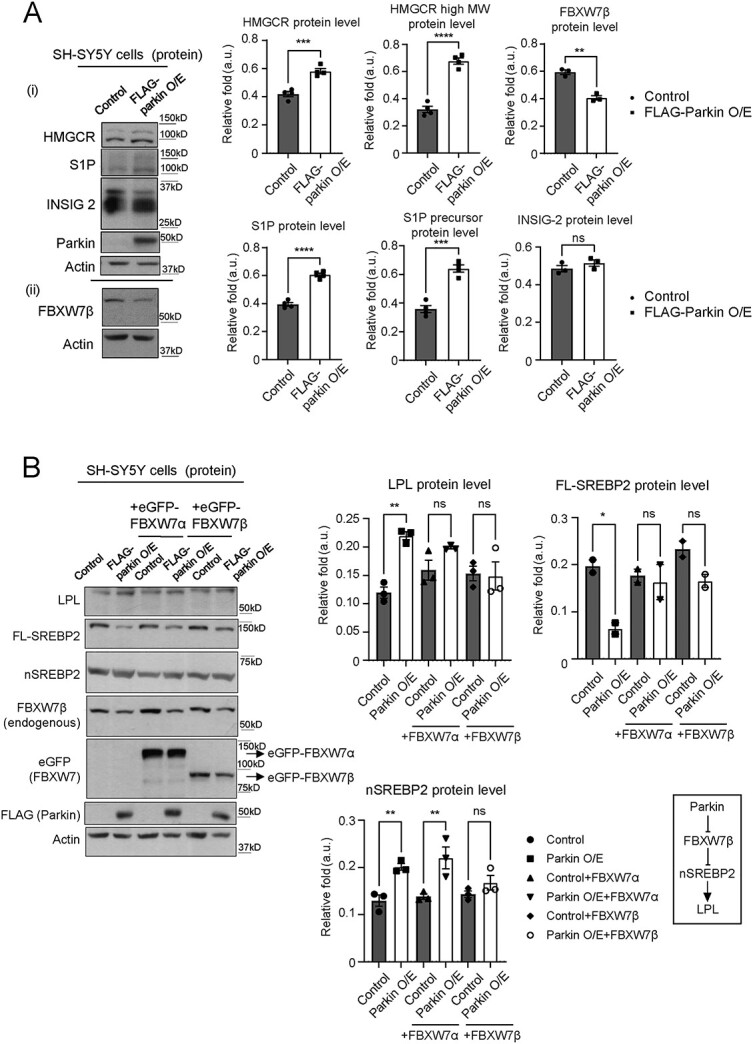
Parkin regulates SREBP2-LPL pathway by downregulating FBXW7β. (**A**) Expression levels of proteins known to be involved in SREBP2 signaling in SH-SY5Y cell line stably overexpressing FLAG-Parkin compared to cells with vector controls. The higher and lower MW bands in S1P blot correspond to precursor and active S1P protein, respectively. (n.s. = not statistically significant; ^*^^*^  *P* < 0.01; ^*^^*^^*^P < 0.001; ^*^^*^^*^^*^P < 0.0001 (*t*-test), *n* = 4 biological replicates for all proteins, except FBXW7β and INSIG-2 (*n* = 3 biological replicates)). Representative images in (i) and (ii) are from independent experiments. Experiment (ii) are identical with the controls in Fig. 3B (lane 1–2). (**B**) Protein expression levels of LPL (detected with LPL 5D2 antibody) and SREBP2 in stable Parkin O/E or control SH-SY5Y cells transiently transfected with either human EGFP-FBXW7α or EGFP-FBXW7β. Overexpressed FLAG-Parkin and FBXW7 protein isoforms were detected using anti-FLAG or anti-GFP antibody, respectively. (n.s. = not statistically significant; ^*^P < 0.05; ^*^^*^P < 0.01 (ANOVA), *n* = 3 biological replicates for all proteins). Last panel shows a hypothetical pathway for Parkin-FBXW7β-SREBP2-LPL signaling. Actin serves as total protein loading controls for (A-B).

Transcriptional activation of SREBP2 target genes requires proteolytic cleavage of the endoplasmic reticulum (ER)-resident, FL-SREBP2 into its active form (i.e. nSREBP2) that translocates to the nucleus upon its activation (33,34). In line with the notion that Parkin promotes SREBP2 downstream signaling, our immunoblotting results revealed that Parkin O/E results in a significant increase in nSREBP2 protein level with a corresponding decrease in precursor FL-SREBP2 protein level compared with SH-SY5Y vector control cells (Fig. 3B and Supplementary Material, Fig. S5). Given that SREBP2 signaling is tightly regulated through various mechanisms (33,34), we then investigate if Parkin increases nSREBP2 level by either: (i) promoting the cleavage of FL-SREBP2 and/or (ii) by preventing the degradation of nSREBP2. As an initial approach, we performed immunoblotting of known SREBP2 pathway regulators such as INSIG-2, Site-1-protease (S1P) and FBXW7 (Fig. 3A and Supplementary Material, Fig. S16). We first confirmed our previous observation that Parkin O/E promotes nSREBP2 signaling as shown by the increase in the protein levels of a SREBP2 downstream target, HMGCR (Fig. 3A). Parkin O/E did not cause any significant change in the expression of INSIG-2, a protein that blocks FL-SREBPs processing (Fig. 3A). Interestingly, we found significant increase in S1P protein levels (higher MW precursor and lower MW active form) in Parkin O/E cells (Fig 3A), suggesting that Parkin may enhance nSREBP2 expression by promoting its proteolytic cleavage via S1P.

On the other hand, nSREBPs are known to be rapidly degraded by the ubiquitin-proteasome pathway via a negative-feedback loop (34). The ubiquitin ligase FBXW7 negatively regulates SREBP2 signaling by enhancing the degradation of nSREBPs upon their phosphorylation by GSK3β (20). Furthermore, Parkin was previously shown to promote the proteasomal degradation of FBXW7 isoform β (FBXW7β) via ubiquitination in neurons (35). Therefore, it is possible that Parkin indirectly promotes the stability of nSREBP2 by enhancing the proteasomal degradation of FBXW7β. Supporting this, we found that stable O/E of familial PD-associated, E3 ubiquitin ligase-deficient Parkin T240R (23,36) induced a significant increase in FBXW7β protein levels and prevented Parkin-induced upregulation of nSREBP2 and LPL in SH-SY5Y cells (Supplementary Material, Fig. S7). Although FBXW7α (MW = ~100 kDa) protein is undetectable in SH-SY5Y cells (Supplementary Material, Fig. S16), our immunoblots revealed a significant reduction of FBXW7β (MW = ~70 kDa) in Parkin O/E cells (Fig. 3A). Moreover, MG-132-mediated proteasomal inhibition was sufficient to normalize both nSREBP2 and FL-SREBP2 expression in Parkin O/E cells to levels that are comparable with control cells (Supplementary Material, Fig. S5). We also established that Parkin O/E did not affect FBXW7 transcript levels (Supplementary Material, Fig. S6). Importantly, O/E of exogenous eGFP-FBXW7β prevents Parkin O/E effect on LPL, FL-SREBP2 and nSREBP2 protein levels (Fig. 3B and Supplementary Material, Fig. S8 and Fig. S16). Collectively, these data demonstrate that Parkin positively influences nSREBP2 level by preventing its degradation via FBXW7β and, possibly, by also promoting its cleavage via S1P.

### Parkin-LPL pathway regulates intracellular LDs deposition

**Figure 4:**
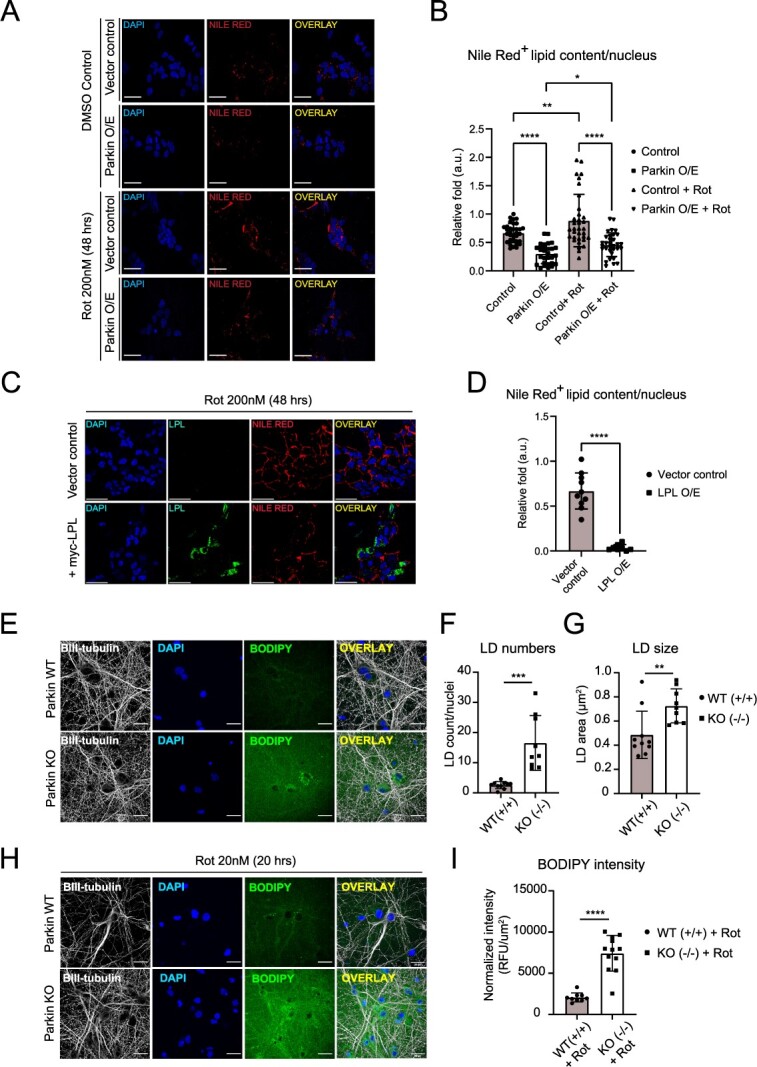
Parkin and LPL regulate intracellular lipid deposition at basal state and during rotenone-induced oxidative stress. (**A**,**B**) Nile Red staining in SH-SY5Y cells stably overexpressing FLAG-Parkin compared to vector controls with or without rotenone (200 nM) treatment for 48 h. (^*^P < 0.05; ^*^^*^P < 0.01; ^*^^*^^*^^*^P < 0.0001 (ANOVA); Control (33 fields); Parkin O/E (29 fields); Control+Rot (34 fields); Parkin O/E + Rot (32 fields), *n* = 3 biological replicates). (**C,D**) Nile Red staining in SH-SY5Y cells transiently expressing myc-LPL compared with vector controls. Both samples were treated with rotenone (200 nM) for 48 h (^*^^*^^*^^*^P < 0.0001 (*t*-test); vector control (10 fields); LPL O/E (8 fields) *n* = 3 biological replicates). LPL was stained with anti-LPL antibody (green). The amount of Lipid droplet-/LD-associated puncta was measured from Nile-Red-positive lipid content. The average signal intensity of Nile Red-positive lipid content per imaging field was normalized to the intensity of control condition (for A–D). (**E**) BODIPY 493/503 staining in Parkin WT (+/+) and Parkin KO (−/−) mouse primary cortical neurons DIV12. Neuronal LDs were identified as BODIPY 493/503-positive, intracellular round-shaped structures. Neurons and cell nuclei were labelled with anti-βIII-tubulin antibody and DAPI respectively. (**F–G**) Quantification of LD number per nuclei and individual LD sizes across conditions as in (E). All measurements were performed for βIII-tubulin-positive cells only. (^*^^*^P < 0.01; ^*^^*^^*^P < 0.001 (*t*-test); Parkin WT (52 cells, 10 fields); Parkin KO (39 cells, 9 fields); *n* = 2 biological replicates). (**H**) BODIPY 493/503 staining in Parkin WT (+/+) and Parkin KO (−/−) mouse primary cortical neurons DIV12 treated with 20 nM rotenone for 20 h. (**I**) Quantification of BODIPY 493/503 fluorescence intensity in neuron samples from (H). Data were normalized to total βIII-tubulin-positive neuronal areas. (^*^^*^^*^P < 0.001 (*t*-test); Parkin WT + Rot (24 cells, 9 fields); Parkin KO (57 cells, 12 fields); *n* = 2 biological replicates). Error bars represent standard deviation between each imaging regions/fields for Fig. 4 B, D, F, G, I. Cell nuclei were stained with DAPI (blue). Scale bar = 20 μm for Fig. 4 E, H.

Recent evidence suggests a strong link between intracellular LD accumulation and cell survival (17,37,38). Notably, LPL activity has been reported to modulate FA uptake and promote intracellular LD deposition (39,40). Accordingly, we performed fluorescence imaging to visualize intracellular lipid deposition via lipid-specific Nile Red dye in SH-SY5Y cells with Parkin O/E. Interestingly, our data show that Parkin O/E cells exhibit significantly lower Nile Red staining intensity compared with control cells at the basal state (Fig. 4A and B). We also treated these cells with 200 nM rotenone, a mitochondrial complex I inhibitor that is linked to PD, to induce oxidative stress. Rotenone treatment elevated intracellular accumulation of Nile Red-positive puncta, which are reminiscent of LDs, in both control and Parkin O/E cells (Fig. 4A and B). This observation is consistent with previous reports in which rotenone treatment induced intracellular LDs accumulation in a glia–neuron co-culture system (16,37). However, the total amount of Nile Red-positive LD-associated puncta is markedly lower in Parkin O/E cell compared with control cells, indicating that Parkin may inhibit excessive LD accumulation during oxidative stress. To test if this phenomenon is mediated by Parkin’s effect on LPL, we transiently O/E myc-LPL in control SH-SY5Y cells and measured the amount of intracellular Nile Red-positive puncta. Similar to Parkin O/E cells, the amount of Nile Red-positive LD-associated puncta is significantly reduced in myc-LPL O/E cells compared with untransfected controls upon rotenone treatment (Fig. 4C and D). Notably, we also observed a substantial colocalization of myc-LPL (~60%) with ER structure (Supplementary Material, Fig. S9), which is an important site of LDs biogenesis (41). Collectively, these observations suggest that Parkin-LPL pathway regulates intracellular LD-associated deposition at basal condition and during PD-linked oxidative stress.

We next investigated whether Parkin regulates LD accumulation in primary cortical neurons. For this experiment, BODIPY 493/503 dye was employed to identify LDs since this dye exhibit more specificity in labelling neutral lipids (the main LD component) compared with Nile Red. Fluorescence imaging revealed that Parkin KO neurons exhibit higher number of BODIPY-positive puncta compared with Parkin WT neurons (Fig. 4E and F). In addition, LD size was also significantly increased in Parkin KO neurons compared with WT neurons (Fig. 4G). Next, we treated both Parkin WT and Parkin KO neurons with rotenone to investigate the role of Parkin in neuronal LD formation under PD-linked oxidative stress. We used a lower dose of rotenone in primary neuron cultures (i.e. 5–100 nM) since the dose used in SH-SY5Y cells (200 nM) rapidly induced neuronal death. Our immunoblot data revealed a significant increase in the level of LD-associated protein Plin2 in primary WT neurons upon acute rotenone treatment (Supplementary Material, Fig. S10). Notably, rotenone-treated Parkin KO neurons exhibit high level of BODIPY fluorescence intensities in both intracellular and extracellular compartments (Fig. 4H). The appearance of large BODIPY-positive structures in Parkin KO neurons hampered accurate quantification of LD puncta in these cells. Thus, we quantified average BODIPY intensity from these cells as a measure of intracellular neutral lipid accumulation. This measurement revealed that Parkin genetic ablation in neurons results in a dramatic increase in intracellular lipid deposition under oxidative stress (Fig. 4I). Altogether, our findings indicate that the loss of Parkin induces aberrant lipid homeostasis and subsequent LD accumulation in neurons under basal condition and in the presence of PD-associated oxidative stress.

### PD-associated oxidative stress triggers the upregulation of Parkin-SREBP2-LPL pathway in neurons

**Figure 5:**
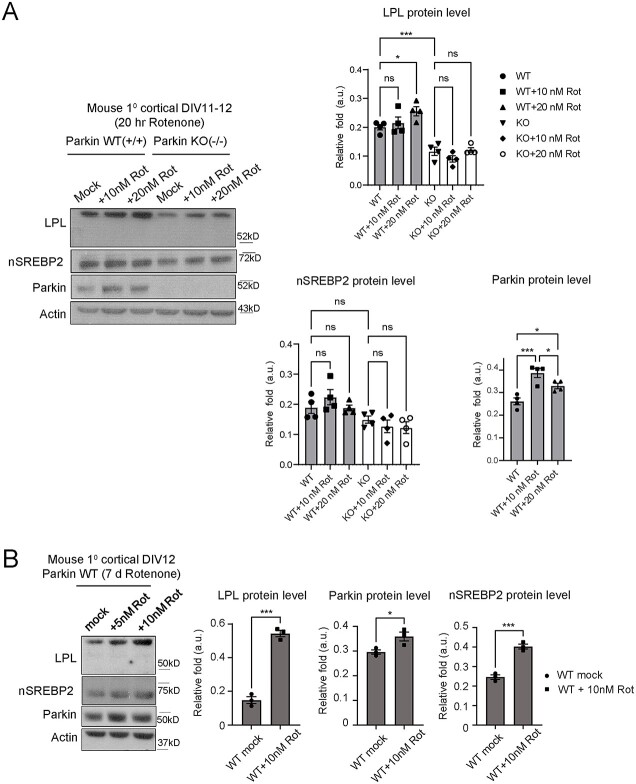
Neuronal LPL, nSREBP2 and Parkin are upregulated upon rotenone-induced oxidative stress. (**A**) LPL (detected with LPL A.4 antibody), nSREBP2 and Parkin protein expression levels in Parkin WT (+/+) and Parkin KO (−/−) mouse primary cortical neurons DIV11-12 treated with 0, 10 or 20 nM rotenone for 20 h. (n.s. = not statistically significant; ^*^P < 0.05; ^*^^*^^*^P < 0.001 (ANOVA), *n* = 4 biological replicates). (**B**) LPL, nSREBP2 and Parkin protein expression levels in Parkin WT (+/+) cortical neurons DIV12 treated with 0, 5 or 10 nM rotenone for 7 days (^*^P < 0.05; ^*^^*^^*^P < 0.001 (*t*-test); *n* = 3 biological replicates for 10 nM rotenone-treated samples). Actin serves as protein loading controls.

Exposure of neurons to oxidative stress, including rotenone treatment, is known to elevate Parkin expression (42,43). On the other hand, activation of SREBP due to either increase in reactive oxygen species (ROS) or mitochondrial defects has been shown to cause glial LD accumulation and neurodegeneration in *Drosophila* photoreceptor system (44). Along the same line, we found that the increase in LD deposition in rotenone-treated (100 nM for 24 h) primary neuron culture correlates with increase in cell death (measured by Plin2 protein and cleaved PARP level respectively; Supplementary Material, Fig. S10). Interestingly, LPL is also significantly elevated upon this high dose rotenone treatment (Supplementary Material, Fig. S10). Given our observations thus far, it is attractive to propose that neurons utilize Parkin-SREBP2-LPL pathway to regulate intracellular, lipid-based protective response against PD-relevant oxidative stress. To test this hypothesis, we treated primary cortical neurons with rotenone (10 nM or 20 nM for 20 h) and measured Parkin, LPL and nSREBP2 protein levels. We found that both Parkin and LPL levels were apparently elevated in Parkin WT neurons upon acute exposure to rotenone at 20 nM concentration (Fig. 5A and Supplementary Material, Fig. S17), whereas the increase in nSREBP2 did not approach statistical significance (Fig. 5A). The observed increase in LPL and Parkin level in Parkin WT neurons was replicated under chronic rotenone treatment (7 days; Fig. 5B). Under this condition, nSREBP2 expression becomes significantly upregulated (Fig. 5B and Supplementary Material, Fig. S17). In contrast, LPL and nSREBP2 levels in Parkin KO neurons remains unaffected when treated with rotenone for up to 20 h (Fig. 5A). Correlating with these observations, we observed a reduction in FBXW7β expression in rotenone-treated WT neurons but not in Parkin KO neurons (Supplementary Material, Fig. S11). Moreover, consistent with previous report (35), FBXW7β protein level was apparently upregulated in Parkin KO neurons at basal state relative to its WT counterparts, a phenomenon that persisted in the presence of rotenone treatment (Supplementary Material, Fig. S11). Taken together, our results show that rotenone treatment induces Parkin-dependent expression of neuronal LPL and nSREBP2. Thus, Parkin-SREBP2-LPL pathway can be activated during neuronal response against oxidative stress, possibly, as a cellular defence mechanism.

### Inhibition of LPL or SREBP2 exacerbates cell death upon exposure to PD-associated oxidative stress

**Figure 6:**
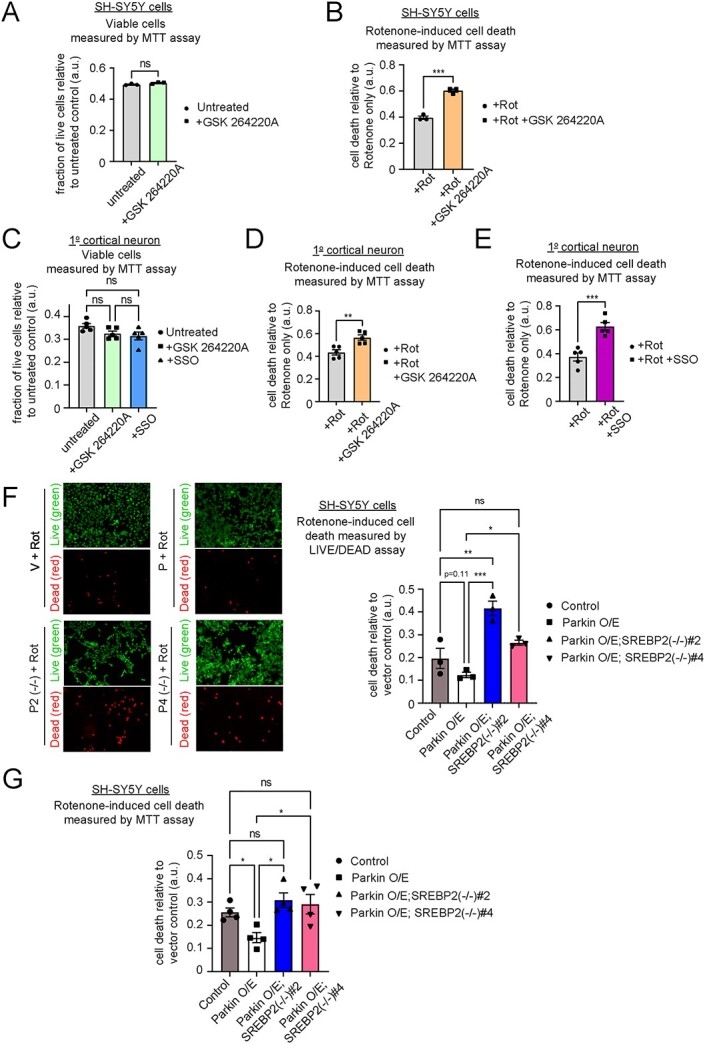
Inhibition of LPL or SREBP2 enhances rotenone-induced cell deaths. (**A**) Cell viability of control SH-SY5Y cells grown in galactose-supplemented media with or without LPL inhibitor (10 μM GSK264220A for 16 h) as measured by MTT assay (**B**) Proportion of rotenone-induced cell death measured by MTT assay in control SH-SY5Y cells grown in galactose-supplemented media treated with rotenone (50 nM for 16 h) alone or co-treated with 10 μM GSK264220A (For (**A,B**) n.s. = not statistically significant; ^*^^*^^*^P < 0.001 (*t*-test), *n* = 3 biological replicates). (**C**) Cell viability of 1^o^ mouse cortical neurons DIV12-15 treated with LPL inhibitor (10 μM GSK264220A) or CD36 inhibitor (10 μM SSO) as measured by MTT assay (**D,E**) Proportion of rotenone-induced cell death measured by MTT assay in 1^o^ mouse cortical neurons DIV12-15 treated with rotenone (50 nM for 24 h) alone or co-treated with (**D**) 10 μM GSK264220A or (**E**) 10 μM SSO. (For (**C–E**) n.s. = not statistically significant (ANOVA); ^*^^*^P < 0.01; ^*^^*^^*^P < 0.001 (*t*-test), *n* = 5 biological replicates). (**F,G**) Rotenone-induced cell death of control cells versus stable Parkin O/E SH-SY5Y cells with or without SREBP2 expression (i.e. Parkin O/E; SREBP2(−/−)#2 and -#4 cells) grown in galactose-supplemented media and treated with 50 nM rotenone for 16 h. (**F**) Cell death as measured by LIVE/DEAD cell viability assay where green fluorescent (calcein-AM) indicates live cells, whereas red fluorescent (ethidium homodimer-1) indicates dead cells. The extent of rotenone-induced cell death is normalized to that of vector control. (n.s. = not statistically significant; ^*^P < 0.05; ^*^^*^P < 0.01; ^*^^*^^*^P < 0.001 (ANOVA), *n* = 3 biological replicates). (**G**) Proportion of rotenone-induced cell death as measured by MTT colorimetric signals normalized to that of vector control (^^*^^P < 0.05 (ANOVA), *n* = 4 biological replicates).

Next, we investigated if LPL inhibition is detrimental to neuronal cells under PD-linked oxidative stress. As a cancer-derived cell line, undifferentiated SH-SY5Y cells rely primarily on aerobic glycolysis (i.e. Warburg effect), instead of mitochondrial oxidative phosphorylation/OXPHOS (45). Thus, we only observed modest cell death upon rotenone treatment, even at high drug concentration (~a few μM for 24–48 h), in these cells (data not shown). To increase the reliance of SH-SY5Y cells on mitochondria OXPHOS, we switched the growth media from glucose supplemented- to galactose-supplemented-DMEM for our subsequent experiments (46). Under this condition, we then measured the extent of rotenone-induced cell death in SH-SY5Y cells treated with an LPL inhibitor (GSK264220A, 10 μM for 16 h) via MTT assay (Fig. 6A and B). Our data show that treatment with the LPL inhibitor alone at the given doses does not affect SH-SY5Y cell or primary cortical neuron viability (Fig. 6A and C). However, co-treatment of GSK264220A with rotenone (50 nM for 16 h) significantly reduced cell viability compared with cells that were treated with rotenone alone (Fig. 6B). Importantly, we observed a similar exacerbation in rotenone- induced cell death in mouse primary cortical neurons that were co-treated with either GSK264220A or a CD36 inhibitor (SSO 10 μM for 16 h), indicating a potential role of LPL and its partner CD36 in PD-relevant neuronal death (Fig. 6D and E).

To determine the potential involvement of SREBP2 signaling in Parkin-mediated neuroprotection against mitochondrial ROS-induced cell death, we then measured the cell viability of Parkin O/E SH-SY5Y cells with or without SREBP2 KO upon rotenone treatment. We first measured the extent of rotenone-induced cell death via a fluorescence imaging approach using LIVE/DEAD cell viability assay (Invitrogen™), whereby green fluorescence (calcein-AM) indicates live cells with intact intracellular esterase activity and red fluorescence (ethidium homodimer-1) indicates dead cells with loss of plasma membrane integrity. As expected from Parkin’s neuroprotective function, Parkin O/E cells display a trend towards reduced cell death upon rotenone treatment (50 nM for 16 h) compared with vector control (Fig. 6F). However, loss of SREBP2 in Parkin O/E cells enhanced the extent of rotenone-induced cell death to a comparable level with that of vector control (Fig. 6F). Moreover, a similar observation was also observed using MTT-based cell viability assay (Fig. 6G), suggesting that SREBP2 is potentially involved in Parkin-mediated cellular protection against rotenone toxicity. Supporting this, we observed significant elevation of mitochondrial ROS level (measured from MitoSOX intensity) in Parkin O/E; SREBP2 KO SH-SY5Y cells compared with control and Parkin O/E parental line at basal state and upon rotenone treatment (Supplementary Material, Fig. S12). Taken together, our data supports a potential role of SREBP2-LPL pathway in Parkin-mediated cellular protection against PD-linked stress, such as mitochondrial ROS generated by rotenone.

## Discussion

In this study, we found that Parkin regulates the expression and activity of LPL in neurons, an effect that is dependent on Parkin’s positive regulation of SREBP2 signaling. Furthermore, we demonstrate that Parkin-SREBP2-LPL axis is activated upon cellular exposure to PD-associated oxidative stress (induced by rotenone), which apparently influences the abundance of LDs—cellular organelles that are thought to be involved in the intrinsic protective mechanism against cellular stress. Importantly, our data show that pharmacological inhibition of LPL exacerbates rotenone-induced cell death. Alongside this, we also demonstrate that Parkin’s protective effect against rotenone-induced cell death is prevented by the genetic ablation of SREBP2. Taken together, our findings highlight a novel role of Parkin in neuronal lipid regulation that may be relevant to PD pathogenesis.

Parkin has previously been implicated in lipid metabolism by promoting the stability of the lipid transporter CD36 that regulates free FA uptake (19). Interestingly, recent metabolomic studies using blood plasma or skin fibroblasts from PD patients with *PARK2* (Parkin) mutations revealed alterations in lipid profiles compared with those of healthy controls (47,48). Here, we show that Parkin positively regulates the expression level of neuronal LPL, a key triglyceride (TG) lipase that controls extracellular free FA uptake via CD36 (22). LPL has been shown to be widely expressed in the brain, including in neurons and glial cells (49). Notably, a recent study demonstrated that circulating brain TG modulates DA transmission in the food reward-associated meso-corticolimbic circuitry via LPL (50). Given the above, it is tempting to speculate that a similar lipid modulation by LPL may be involved in nigrostriatal DA neurons responses against PD-linked neurodegenerative insults. Supporting this, we showed in this study that Parkin-dependent upregulation of LPL in neuronal cells is associated with LD homeostasis under PD-associated oxidative stress.

Although current evidence for LPL’s role in neuronal survival is lacking, there is scientific evidence to support a role for SREBP signaling in neurodegeneration. In a recent study, Chali *et al*. (51) reported that excitotoxic neurodegeneration induced by *in vivo* kainate injection in mouse hippocampus acutely promotes nuclear SREBP2 translocation and cholesterol synthesis. In another study, Liu *et al*. (44) demonstrate that SREBP activation in photoreceptor neurons promotes LD accumulation in surrounding glial cells and subsequent neurodegeneration in *Drosophila* photoreceptor system in the presence of high level of ROS. In the context of PD, inhibition of Stearoyl-CoA desaturase 1 (SCD1), which is a downstream target of SREBP1, seems sufficient to suppress α-Synuclein induced-toxicity in yeast and human neurons (15,52). Interestingly, a web-based genome-wide association study has identified a mutation in *SREBF1* locus as a genetic risk factor for sporadic PD in a European population (6). In this study, we found that SREBP2 signaling is upregulated in primary cortical neurons upon exposure to Rotenone (Fig. 5B). Parkin itself seems to be important in promoting nSREBP2 signaling, since Parkin O/E elevated nSREBP2 protein level in SH-SY5Y cells (Figs. 2E and 3B) and Parkin genetic ablation prevents Mevastatin-induced pharmacological activation of SREBP2 in primary neurons (Fig. 2C). Therefore, Parkin-dependent SREBP2 signaling is likely to play an important role in PD-associated neurodegeneration process.

Parkin may regulate SREBP2 signaling through the following scenarios: (i) promoting maturation of FL-SREBP2 to nSREBP2; (ii) preventing degradation of nSREBP2 and/or (iii) enhancing the transcription of *SREBF2* gene. For the first scenario, our data show that Parkin O/E increases the level of S1P, one of the Golgi-resident proteases that cleaves FL-SREBP2 to its nuclear form upon pathway activation (34). Notably, the protein stability of FL-SREBP2 regulators, such as SCAP and INSIGs, are regulated via ubiquitination by other E3 ligases (53–56). Therefore, the possibility that Parkin directly regulates S1P protein stability via its E3 ligase activity warrants further investigation. For the second scenario, our data demonstrate that Parkin O/E downregulates the expression level of FBXW7β, a component of SCF^Slimb^ E3 ligase complex that enhances degradation of nSREBPs upon their phosphorylation by GSK3β (20). Of note, we found that only FBXW7β, but not FBXW7α and -γ, was expressed by SH-SY5Y cells. Moreover, our immunofluorescence data demonstrate that exogeneous eGFP- FBXW7β is localized in both cytoplasm and nuclear compartment of the cells (Supplementary Material, Fig. S8). These observations suggest that FBXW7β may substitute the role of FBXW7α and -γ in these cells and this may explain the discrepancy between our data and those of Sundqvist *et al* study (20). The Parkin-dependent regulation of FBXW7β observed here is in an agreement with a previous study showing that Parkin promotes the proteasomal degradation of FBXW7β via ubiquitination in neurons (35). Moreover, exogenous expression of eGFP-FBXW7β (Fig. 3B) or elevation of endogenous FBXW7β due to E3-ligase-deficient Parkin T240R O/E (Supplementary Material, Fig. S7) is sufficient to negate Parkin O/E effect on SREBP2 and LPL levels. Therefore, our data support the notion that Parkin promotes nSREBP2 stability by promoting the degradation of its negative regulator FBXW7β. Finally, our data show that Parkin level positively correlates with *SREBF2* transcript level (Fig. 2A and B). Along this line, Parkin has been demonstrated to act as a transcription factor that promotes neuroprotection by downregulating p53 transcript through its DNA binding activity (57,58). Thus, future study should also investigate the possibility of Parkin acting as a transcription factor for *SREBF2* gene expression.

LD-associated structures have been observed in several neurodegenerative diseases (38,59,60). In PD, lipid-containing structures have been shown to be one of the main components of LBs found *in vitro* (12) and PD patient brains (11). Interestingly, O/E of α-Synuclein was sufficient to trigger LD accumulation and cellular toxicity in yeast and neuronal PD models (13,15). Our findings demonstrate that rotenone-induced oxidative stress also triggered LD deposition, which was accompanied with increase in cell death (Fig. 4 and Supplementary Material, Fig. S10). Since increase in LPL expression reduces intracellular lipid deposition during rotenone-induced stress, we posit that the activation of Parkin-SREBP2-LPL pathway might represent a novel cellular protective mechanism against PD-associated stress. Indeed, our results demonstrate that pharmacological inhibition of LPL by GSK264220A enhances the extent of rotenone-induced cell death in SH-SY5Y cells and primary cortical neuron compared to those treated with rotenone alone. Moreover, genetic ablation of SREBP2 elevates mitochondrial ROS (Supplementary Material, Fig. S12) and abrogates cellular protection conferred by Parkin O/E upon rotenone treatment (Fig. 6F and G). Therefore, our results strongly support that activation of Parkin-SREBP2-LPL pathway in neuronal cells is important for mitigating cellular toxicity against PD-relevant stress.

Increase in LPL activity has been shown to increase extracellular free FA uptake via CD36 (22,61) and promotes LD accumulation in peripheral cells (39). In contrast, our findings revealed that Parkin-dependent increase in LPL level resulted in lower LD deposition in SH-SY5Y cells, whereas loss of Parkin leads to increased LD accumulation in primary cortical neurons at basal state and under oxidative stress. Therefore, LPL may exhibit non-canonical role in neuronal LD maintenance under certain conditions (i.e. Parkin activation or oxidative stress). Interestingly, early studies on LPL biology had reported detectable intracellular LPL activities in various study models (62,63). Consistent with this, our immunofluorescence imaging data suggest that a substantial fraction of exogenously expressed myc-LPL was colocalized with ER (Supplementary Material, Fig. S9), the primary site of LD biosynthesis (41). Altogether, these results suggest a non-conventional role of intracellular LPL in regulating LD homeostasis in a PD-relevant context.

In summary, our study shows that Parkin promotes SREBP2 signaling by elevating basal nSREBP2 level. In turn, increase in SREBP2 activity enhances LPL expression and activity. In neuronal cells, LPL activity regulates intracellular LD deposition at basal condition and under oxidative stress. In the latter scenario, LD accumulation may initially promote cell viability by sequestering free FAs from harmful oxidation, assisting mitochondrial functions and/or provides lipid materials for autophagy process (17,38). However, excessive LD build-up and elevated peroxidated lipid level can cause detrimental effects to neurons at the later stage, such as promoting formation of insoluble α-Synuclein inclusions and inhibiting mitophagy (14,15). These events will in turn lead to toxicity cascades leading to degeneration of neurons, especially those that are vulnerable to metabolic insults, such as DA neurons (Supplementary Material, Fig. S13). Under these conditions, Parkin-SREBP2-LPL pathway is activated to suppress excessive LD formation and dysfunction of this molecular pathway may contribute to neurodegeneration seen in PD. We foresee that our study will advance our understanding on the role of dysregulated lipid homeostasis in PD pathogenesis and aid subsequent development of new therapeutic strategy against this debilitating disease.

## Materials and Methods

### SH-SY5Y cell cultures

Human SH-SY5Y neuroblastoma cells (ATCC) were grown in Dulbecco’s Modified Eagle’s medium (DMEM, high glucose; Sigma-Aldrich, MO, USA), with the supplementation of 10% fetal bovine serum (FBS; Gibco, USA) and 100 units/ml Penicillin–Streptomycin (Gibco, USA). The cells were all maintained in 5% CO_2_ at 37°C. For transfection experiments, cells were plated on 6-well plates and 100-mm dishes (for immunoprecipitation experiments) and transiently transfected (at 60–70% confluency) with various desired plasmids (Table 1) using Lipofectamine PLUS reagent (Invitrogen, CA, USA) according to the manufacturer’s protocol. Cells were harvested 40–48 h after transfection for further analyses. Our laboratory has previously generated stably over-expressing FLAG-Parkin (Wild-type) cells line (PK7) and FLAG-Parkin T240R (PD-associated mutant) alongside a control line expressing empty vector [V4; (23)]. For experiments with pharmacological agents, rotenone (Sigma-Aldrich, USA), Mevastatin (Sigma-Aldrich, USA), MG-132 (AG Scientific, CA, USA), GSK264220A (Tocris, UK) and Sulfo-*N*-succinimidyl Oleate/SSO (Sigma-Aldrich, USA) were dissolved in DMSO (Sigma-Aldrich, USA or FisherMPB, France) and stored at −20°C prior to usage. For rotenone-induced cell death measurements in SH-SY5Y cells (Fig. 6), we used identical DMEM medium without glucose (Sigma-Aldrich, MO, USA) and supplemented with 10 mm galactose (Sigma-Aldrich, MO, USA).

**Table 1:** List of plasmids

Plasmid name	Source & reference
pEGFP-Parkin	Addgene#45875 was a gift from Edward Fon (64)
pSpCas9(BB)-2A-Puro (PX459) V2.0	Addgene#62988 was a gift from Feng Zhang (31)
pcDNA-myc-LPL	Generated in this study (see Material and Methods)
pcDNA3.1-2xFLAG-SREBP-2	Addgene#26807 was a gift from Timothy Osborne (29)
pLKO-puro FLAG SREBP2	Addgene#32018 was a gift from David Sabatini (30)
pEGFP-FBXW7α	Generated in this study (see Material and Methods)
pEGFP-FBXW7β	Generated in this study (see Material and Methods)

### Generation of pcDNA-myc-LPL, pEGFP-FBXW7α and pEGFP-FBXW7β constructs

Prior to cloning LPL, human cDNA library was obtained by first performing an RNA extraction from SH-SY5Y cells using the RNA isolation kit as per manufacturer protocol (Qiagen, Germany). The RNA was then reverse transcribed, by using SuperScriptII Reverse Transcriptase (Invitrogen, CA, USA), following which, a PCR was performed using primers specific to human LPL, FBXW7α or FBXW7β cDNA. LPL cDNA was then cloned into myc-pcDNA using BamHI and XhoI restriction enzyme (NEB, USA) sites. The resulting constructs generate myc-tagged LPL at N-terminus. Meanwhile, FBXW7 cDNAs were cloned into pEGFP-Parkin WT backbone (Addgene #45875) by swapping the pre-existing Parkin cDNA with either FBXW7α or FBXW7β cDNA using EcoRI and ApaI restriction enzyme (NEB, USA) sites. The resulting constructs generate EGFP-tagged FBXW7 at N-terminus.

### Generation of CRISPR-mediated SREBP2 KO SH-SY5Y cell lines

Two gRNAs targeting exon 3 and exon 15 of human SREBF2 were designed using Benchling CRISPR design tools (https://www.benchling.com/). The targeting sequences of the gRNAs are as follows: (i) gRNA SREBF2 exon 3: *5*′ *GAACTGTCTGCACCGTAGCCG 3′*; (ii) gRNA SREBF2 exon 15: *5*′ *GTGGACGTCTGCAATCATG 3′*. The two gRNAs were cloned individually to pSpCas9(BB)-2A-Puro (PX459) plasmid (Table 1) using BbsI restriction enzyme cut sites. To generate stable SREBP2 (−/−) KO SH-SY5Y lines, SH-SY5Y V4 and PK7 cell lines were transiently co-transfected with PX459 gRNA SREBF2-exon 3 and -exon 15. Thereafter, positively transfected cells were isolated using puromycin (1 μg/ml) selection and seeded individually in 96 well plates for genomic screening. Positive clones that harbor homozygous deletion of exon 3–15 of SREBF2 gene (SREBF2 Δ ex3–15) were identified by the PCR products of genomic DNA using the following primers: (i) Forward primer (SREBF2 exon 3): *5*′ *TCCTTCAGCCTCAAGTCCAAAGCC 3′*; (ii) Reverse primer (SREBF2 intron 3): *5*′ *GCCCTACAAGCTGCTTTGTGTACACT3*′; (iii) Reverse primer (SREBF2 intron 15): *5*′ *TGCTGGCCCAACTTGAGTTCTCC 3′*.

### Parkin KO mouse models

C57BL/6J mouse model harboring deletion of exon 7 of Parkin gene was previously described (21). For the experiments, two homozygous mouse lines (i.e. Parkin (+/+) WT and Parkin (−/−) KO lines) were generated by crossing the initial heterozygous Parkin Δexon7/+ founder mice and maintained in homozygous genotype throughout the study. Mouse genotyping was performed according to the previous study (21). Only male mice were used for immunoblotting and LPL hydrolysis assay experiments. All mouse studies were approved and conducted according to the guidelines of the Institutional Animal Care Committee of Tan Tock Seng Hospital-National Neuroscience Institute, Singapore and NTU-LKCMedicine Animal Research Facility.

### Primary mouse cortical neuron

Primary mouse cortical neuron cultures were adapted from previously described protocol (65). Briefly, cortical tissues were dissected from Parkin WT and KO E17-E18 mice brains and their meninges were removed. The tissues were subsequently incubated with papain solution (Worthington, USA) for 45 min, washed and dissociated in DMEM with FBS. The cell suspension was spun down at 250 *g* for 5 min to remove excess media. Cell pellets were resuspended in Neurobasal (NB) media supplemented with 1× B27 and 1× GlutaMAX (NB/B27 media, Gibco, USA) and plated on poly-D-Lysine (PDL, Sigma-Aldrich, USA)-coated tissue culture plates (0.01 mg/ml PDL) or glass coverslips (0.1 mg/ml PDL). Primary neurons were grown NB/B27 media for 11–21 days *in vitro* (DIV). Additional NB/B27 media (1/3 volume) were added at DIV4 to sustain culture growth.

### Preparation of cell lysates and brain homogenates

Prior to lysate collection, SH-SY5Y cells and primary neurons were briefly rinsed with ice-cold DMEM media without FBS or PBS, respectively. Cells were lyses in SDS lysis buffer (PBS with 1% SDS, Phenylmethylsulfonyl Fluoride/PMSF (Sigma-Aldrich, Germany), Aprotinin (Sigma-Aldrich, USA), phosphatase inhibitor cocktail 2 and 3 (Sigma-Aldrich, USA) and collected in Eppendorf tubes. Whole brain tissues were harvested from Parkin WT and KO adult male mice (4–10 months) and homogenized with Dounce homogenizer inside 1% SDS lysis buffer. Both cell and whole brain lysates were sonicated (3 × 10 s, amplitude: 30–40%) and centrifuged at 13 500 rpm at RT for 15 min to remove insoluble fraction before the supernatant was collected. Protein concentrations of cell or whole brain lysates were determined by Bradford protein assay (Bio-Rad, CA, USA). Western/Immunoblotting was performed using 20–40 μg of total protein.

### Immunoblot analysis

Prior to immunoblot analysis, cell lysates were diluted in 2X loading buffer (62.5 mm Tris–HCl (pH 6.8) (Sigma-Aldrich, USA), 20% glycerol (Invitrogen, CA, USA), 2% SDS (Invitrogen, NY, USA), 4% β-mercaptoethanol (Sigma-Aldrich, CA, USA), 0.008% bromophenol blue (Bio-rad, CA, USA)) and boiled at 100°C, 5 min. Proteins were then resolved via SDS-PAGE (BioRad, USA) and transferred onto nitrocellulose (GE Healthcare, USA) membranes at either 100 V for 2 h or 30 V for 16 h. The membranes were then blocked with 5% non-fat milk in TBS-T buffer (25-mm Tris–HCl, *p*H 7.4, 137-mm NaCl, 2.68-mm KCl and 0.05% Tween-20) (Sigma-Aldrich, USA) for at least 1 h at room temperature (RT). The membranes were then incubated with desired primary antibodies (Table 2) and rotated in 4°C overnight. Subsequently, the membranes were washed in TBS-T buffer and incubated with horseradish peroxidase (HRP)-conjugated secondary antibody (Table 2) for 1–2 h at RT. HRP signals, which correspond to protein levels, were detected by various ECL detection reagents (ECL Western Blotting Substrate (Pierce, ThermoScientific, USA), Amersham ECL Western Blotting Detection Reagents (GE Healthcare, USA) and WesternBright Sirius Western Blotting detection kit (Advansta, CA, USA)).

**Table 2:** List of antibodies

Antibody	Host species	Source
LPL (LPLA.4)	Mouse, monoclonal	Abcam, UK (ab21356)
LPL (LPL 5D2)	Mouse, monoclonal	Abcam, UK (ab93898)
SREBP2(ab1,detect nSREBP2)	Rabbit, polyclonal	Abcam, UK (ab112046)
SREBP2 (ab3,detect FLSREBP2)	Rabbit, polyclonal	Abcam, UK (ab30682)
Parkin (PRK8)	Mouse, monoclonal	Cell Signaling Technology, USA (#4211)
CD36	Rabbit, polyclonal	Abcam, UK (ab78054)
EndoL (LIPG)	Rabbit, polyclonal	Abcam, UK (ab24447)
β-actin	Mouse, monoclonal	Sigma-Aldrich, USA (A5441)
Plin2	Rabbit, monoclonal	Abcam, UK (ab108323)
FBXW7	Rabbit, polyclonal	Abcam, UK (ab109617)
S1P	Rabbit, polyclonal	Abcam, UK (ab59870)
INSIG 2	Rabbit, polyclonal	Abcam, UK (ab86415)
HMGCR	Rabbit, monoclonal	Abcam, UK (ab174830)
GFP	Mouse, monoclonal	Roche, Sigma-Aldrich, USA (11814460001)
FLAG-HRP	Mouse, monoclonal	Sigma-Aldrich, USA (A8592)
c-Myc-HRP (9E10)	Mouse, monoclonal	Roche, Sigma-Aldrich, USA (11814150001)
β-Tubulin isotype III (TU-20)	Mouse, monoclonal	Sigma-Aldrich, USA (T5076)
Anti Rabbit-HRP	Donkey	Amersham, Cytiva, UK (NA934)
Anti Mouse-HRP	Sheep	Amersham, Cytiva, UK (NA931)
Anti Rabbit-Rhodamine	Goat	Invitrogen, USA (R6394)
Anti Mouse-Alexa Fluor 647	Donkey	Invitrogen, USA (A31571)

### Immunoprecipitation

SH-SY5Y cells were harvested and subsequently subject to lysed with lysis buffer while on ice. The lysates were then centrifuged at 13 500 rpm, 15 min at 4°C. The supernatant was collected, and a Bradford protein assay was done to determine protein concentration in the lysate. Equal amounts of protein were then used for immunoprecipitation with the desired antibodies and left to incubate at 4°C overnight. Protein G PLUS/protein A-agarose (Calbiochem, Millipore, Merck, USA) was added the following day and left to incubate with the lysates for 2 h at 4°C. Subsequently, the agarose beads were washed with 1% Triton X-100/PBS (Bio-Rad, CA, USA) for a total of 5 times. Proteins captured by the beads were then released by adding SDS. Immunoprecipitates and their respective cell lysates were resolved by immunoblotting, as described previously.

### Reverse transcription (RT) PCR

For cell lines and primary neurons, total RNAs were extracted using Rneasy Mini Kit (Qiagen, Germany) according to manufacturer’s protocol. For whole brain RNA samples, Parkin WT and KO mice brains were firstly flash-freezed in liquid nitrogen. The brains were then homogenized using a mortar and a pestle. Thereafter, RNAs were isolated using TRIzol reagent (Invitrogen, USA) according to the instructions provided by the manufacturer. RNAs from all samples were converted to cDNA using SuperScript II First Strand Synthesis System (Invitrogen, CA, USA) according to the manufacturer’s protocol. Subsequent quantifications of gene transcripts were done by PCR amplification using primers from either Integrated DNA Technologies (Singapore) or KiCqStart SYBR Green primers (Sigma-Aldrich, USA; Table 3). The PCR products were resolved using DNA agarose gel electrophoresis and visualized with XRS+ Gel Documentation system (Bio-Rad, USA).

**Table 3:** List of primers

Gene	Primer sequence
Human LPL (exon 5-7)	Forward Primer: 5′– GAGTCGTCTTTCTCCTGATG—3′ Reverse Primer: 5′– GGTATGGGTTTCACTCTCAG—3′
Human LPL (exon 7-10)	Forward Primer: 5′– TCTGCCTGAAGTTTCCACAAA—3′ Reverse Primer: 5′– GCCCAGTTTCAGCCTGACTT—3′
Mouse LPL	Forward Primer: 5′– GAGACTCAGAAAAAGGTCATC—3′ Reverse Primer: 5′– GTCTTCAAAGAACTCAGATGC—3′
Human SREBF2 (exon 6-7)	Forward Primer: 5′– CAGCAGGTCAATCATAAACTG—3′ Reverse Primer: 5′– GGACATTCTGATTAAAGTCCTC—3′
Mouse SREBF2	Forward Primer: 5′–GAGGCGGACAACACACAATA– 3′ Reverse Primer: 5′–CGGCTCAGAGTCAATGGAATAG– 3′
Mouse SREBF2 (2)	Forward Primer: 5′– TGGTAAATGGTGTGATTGTC– 3′ Reverse Primer: 5′– GATAAGCAGGTTTGTAGGTTG– 3′
Mouse SREBF1	Forward Primer: 5′–GTGTGTACTGGCCTTTCTGTG– 3′ Reverse Primer: 5′–TGTCTCCAGAAGTGTACAGCC– 3′
Mouse ANGPTL3	Forward Primer: 5′–CTCCAGAGTGTGGAAGAACAG– 3′ Reverse Primer: 5′–TCAGTTGAAGAGGGGGAGTAG– 3′
Mouse ABCG1	Forward Primer: 5′–GTCCCTTATCCACTGTCCATAG– 3′ Reverse Primer: 5′–CTGAAGGAACTCACCTTCTAGG– 3′
Human SorLA	Forward Primer: 5′–CCTCTGAAGGTGACCACATAAC– 3′ Reverse Primer: 5′–CTGTCTGTCCCACCCAAATATC– 3′
Human LXRa	Forward Primer: 5′–AACCCTGGGAGTGAGAGTAT– 3′ Reverse Primer: 5′–TTAGCATCCGTGGGAACATC– 3′
Human Actin	Forward Primer: 5′–AGGCCAACCGCGAGAAGATG– 3′ Reverse Primer: 5′– TACCCCTCGTAGATGGGCAC– 3′
Human GAPDH	Forward Primer: 5′–GAAGGTGAAGGTCGGAGTCAACG– 3′ Reverse Primer: 5′– TGCCATGGGTGGAATCATATTGG– 3′
Human Parkin (exon 3-5)	Forward Primer: 5′–GGAAGTCCAGCAGGTAGATCA– 3′ Reverse Primer: 5′–ACCCTGGGTCAAGGTGAG– 3′
Human HMGCR	Forward Primer: 5′– ACTTCGTGTTCATGACTTTC– 3′ Reverse Primer: 5′–GACATAATCATCTTGACCCTC– 3′
Mouse HMGCR	Forward Primer: 5′–GATAGCTGATCCTTCTCCTC– 3′ Reverse Primer: 5′– ATGCTGATCATCTTGGAGAG– 3′
Human DHCR24	Forward Primer: 5′– ACACCAAGAAACAGATTGTC– 3′ Reverse Primer: 5′– TACTTGTGGGATGATGACTC– 3′
Mouse LDLR	Forward Primer: 5′– TGTCCATCTTCTTCCCTATTG– 3′ Reverse Primer: 5′– GTCTTGAGGGGTAGGTATAG– 3′
Human FBXW7 (pan)	Forward Primer: 5′–GGGGATTGATGAACCATTGCAC– 3′ Reverse Primer: 5′–TGTCCTTTCAGCACCTTAGGA– 3′
Human FBXW7α	Forward Primer: 5′–CGTTCACCAACTCTCCTCCC– 3′ Reverse Primer: 5′–CAAGCCCAGTGGTACTTGT– 3′
Human FBXW7β	Forward Primer: 5′–GCACAGAATCACTGAAGGGG– 3′ Reverse Primer: 5′–GCTGAACATGGTACAAGCCC– 3′

### LPL activity assay

LPL lipid hydrolysis activity in mouse brain samples were measured using LPL Activity Fluorometric Assay Kit (Biovision, USA) according to the manufacturer’s protocol.

### Cell viability assays

MTT (MedChemExpress, USA) (5 mg/ml in PBS) was added directly to the cell media to working concentration of 0.3 mg/ml and incubated for 3–4 h. Thereafter, the cells were lysed with pure DMSO and the lysates was transferred to clear bottom 96 well plate. MTT signal was measured at 570-nm wavelength using microplate reader (Tecan). LIVE/DEAD cell viability assay (Invitrogen, USA) was performed according to the manufacturer’s protocol. Live fluorescence imaging was performed after prior incubation with calcein-AM (green fluorescence) and ethidium homodimer-1 (red fluorescence) with an epifluorescence microscope (Olympus IX70). Cell counting was performed with ImageJ.

### MitoSOX live imaging experiment

SH-SY5Y control, Parkin O/E and Parkin O/E;SREBP2(−/−) cells were grown in galactose-supplemented media prior to staining with MitoSOX Red™ (Invitrogen, USA) and rotenone treatment. Cells were co-stained with 5 μM MitoSOX, Hoechst (1:1000; Enzo Life Sciences, USA) and BODIPY 493/503 (Invitrogen, USA) (1:1000) in culture media for 10 min at 37°C. Cells were rinsed and imaged in HBSS without phenol red (Gibco, USA) supplemented with Ca^2+^ and Mg^2+^. Live confocal fluorescence imaging was performed on a spinning disc confocal (SDC) setup built around a Nikon Ti2 inverted microscope equipped with a Yokogawa CSU-W1 confocal spinning head, a Plan-Apochromat objective (100×, 1.45 NA) and a back-illuminated sCMOS camera (Prime 95B; Photometrics, USA). All image acquisitions were carried out using MetaMorph (Molecular Device, USA) with exposure time 500 ms. Automated MitoSOX intensity measurements were done using MatLab algorithm (66) and is available upon request.

### Fluorescence microscopy

Immunofluorescence (IF) imaging was done using paraformaldehyde (PFA )-fixed samples grown on PDL-treated glass coverslips. Briefly, samples were washed with ice-cold PBS and fixed in 4% PFA solution (ChemCruz, TX, USA) at 4°C overnight. Samples were then washed with PBS and permeabilized with 0.1% Triton-X solution in PBS for 5 min. Subsequently, samples were incubated with appropriate primary antibodies (Table 2) in fluorescence dilution buffer (FDB; 5% FBS, 5% goat serum (Millipore, USA) and 2% BSA (Sigma-Aldrich, USA) in PBS) at 4°C overnight. The following day, samples were washed with PBS and incubated with fluorescence-conjugated secondary antibodies (Table 2) for 1 h at RT. For nuclei staining, samples were then incubated with DAPI solution for 5 min, washed, and mounted to glass slides with FluorSave™ mounting reagent (EMD Millipore, USA). For staining of LDs, samples were PFA-fixed, permeabilized with 0.1 mg/ml Saponin (Sigma-Aldrich, USA) and co-stained with primary-secondary antibodies as described previously. Thereafter, samples were additionally incubated with either Nile Red (Invitrogen, USA) or BODIPY493/503 (Invitrogen, USA) for 10–15 min according to published protocol (67). Both IF and dye-stained samples were visualized using Fluoview Upright Confocal Microscope (Olympus FV1000 and FV3000) using 60× or 100× objectives equipped with 405, 488, 543 and 633 nm wavelength gas state lasers. LDs were quantified using an in-house automated image analysis software (Adcount by Nair *et al*, unpublished. The algorithm can be accessed at https://adcount.github.io/) or Matlab algorithm (66), which are available upon request.

### Statistical analysis

All data were expressed as mean with error bar (standard error of the mean) unless otherwise stated. The data was accumulated under each condition from at least two independent experiments. The datasets in Figures 1, 2, 3, 5 and 6 were normalized using ‘normalization by sum of the replicate’ method as it minimizes the issue related to high coefficient of variation (CV) for data with low quantified intensities when ‘normalisation by fixed point’ method is performed (68). The statistical analyses were performed using GraphPad Prism with student’s two-tailed unpaired *t*-test or one-way analysis of variance (ANOVA) with Benjamini–Hochberg procedure to decrease false discovery rate (FDR). The *P* value of < 0.05 is considered as statistically significant.

## Supplementary Material

FigS1_ddac297

FigS2_ddac297

FigS3_ddac297

FigS4_ddac297

FigS5_ddac297

FigS6_ddac297

FigS7_ddac297

FigS8_ddac297

FigS9_ddac297

FigS10_ddac297

FigS11_ddac297

FigS12_ddac297

FigS13_ddac297

FigS14_ddac297

FigS15_ddac297

FigS16_ddac297

FigS17_ddac297

Supplementary_legends_2nd_rev_HMG-2022-CE-00394_R1_ddac297

## Data Availability

The authors confirm that the data supporting the findings of this study are available within the article and its Supplementary material. Raw data that support these findings are available from the corresponding author, upon reasonable request.
