# Correction to: Parkin regulates neuronal lipid homeostasis through SREBP2-lipoprotein lipase pathway—implications for Parkinson's disease

**DOI:** 10.1093/hmg/ddad068

**Published:** 2023-05-02

This is a correction to: Willcyn Tang, John Thundyil, Grace Gui Yin Lim, Teddy J W Tng, Sean Qing Zhang Yeow, Aditya Nair, Chou Chai, Tso-Pang Yao, Kah-Leong Lim, Parkin regulates neuronal lipid homeostasis through SREBP2-lipoprotein lipase pathway–implications for Parkinson's disease, *Human Molecular Genetics*, Volume 32, Issue 9, 1 May 2023, Pages 1466–1482, https://doi.org/10.1093/hmg/ddac297

In the originally published version of this manuscript, in Figure 4A, panels in the bottom row (Rot200nM (48 hrs), Parkin O/E) inadvertently duplicated the panels in the top row (DMSO Control, Vector control).

The Figure has now been corrected.

