# Supplementary figures and images for: Parkin regulates neuronal lipid homeostasis through SREBP2-lipoprotein lipase pathway—implications for Parkinson’s disease
Source: Hum Mol Genet. 2022 Nov 26;32(9):1466–82. doi: 10.1093/hmg/ddac297 (PMC10117165; doi:10.1093/hmg/ddac297)

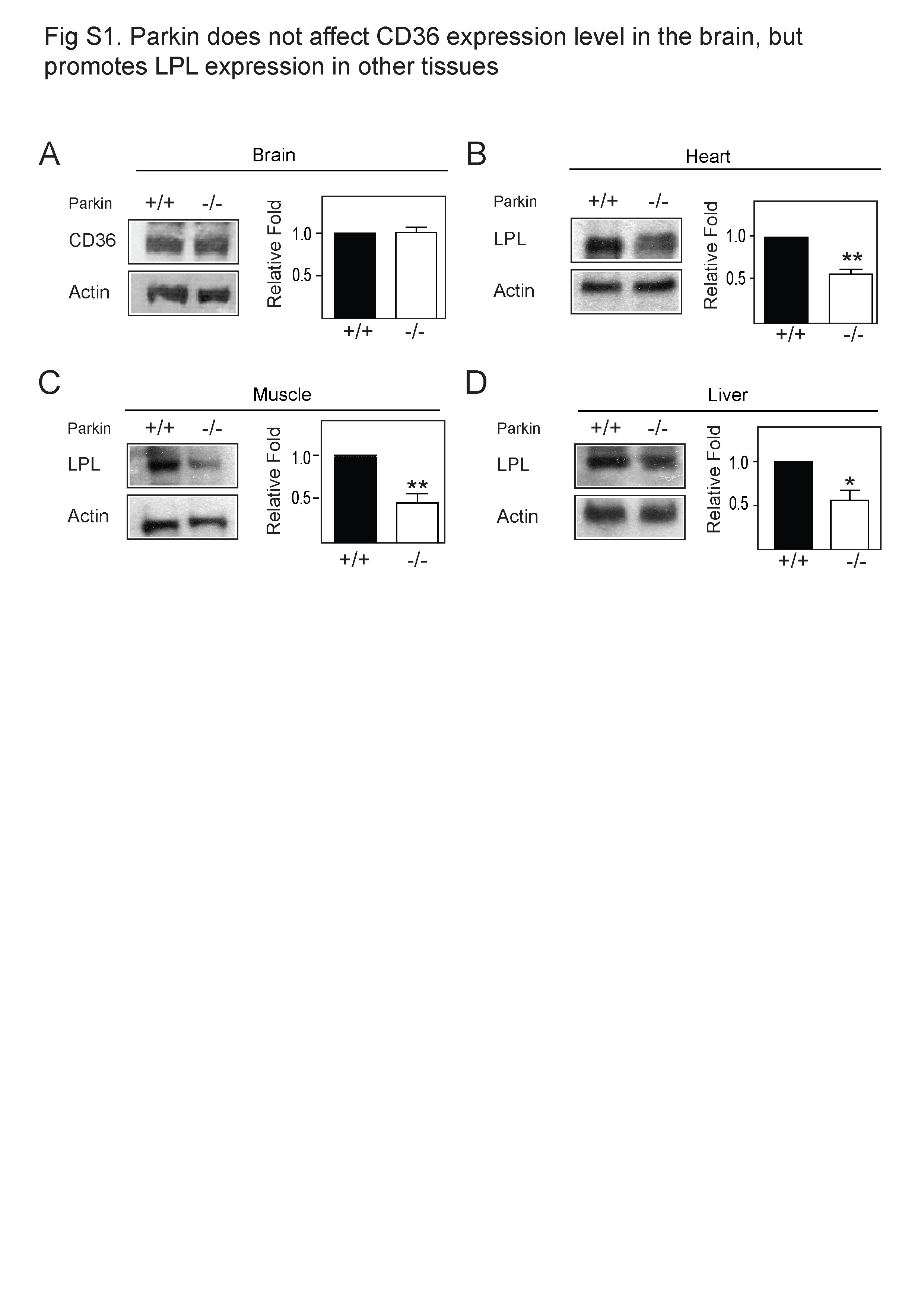

Supplement: FigS1_ddac297 [file figs1_ddac297.jpeg]

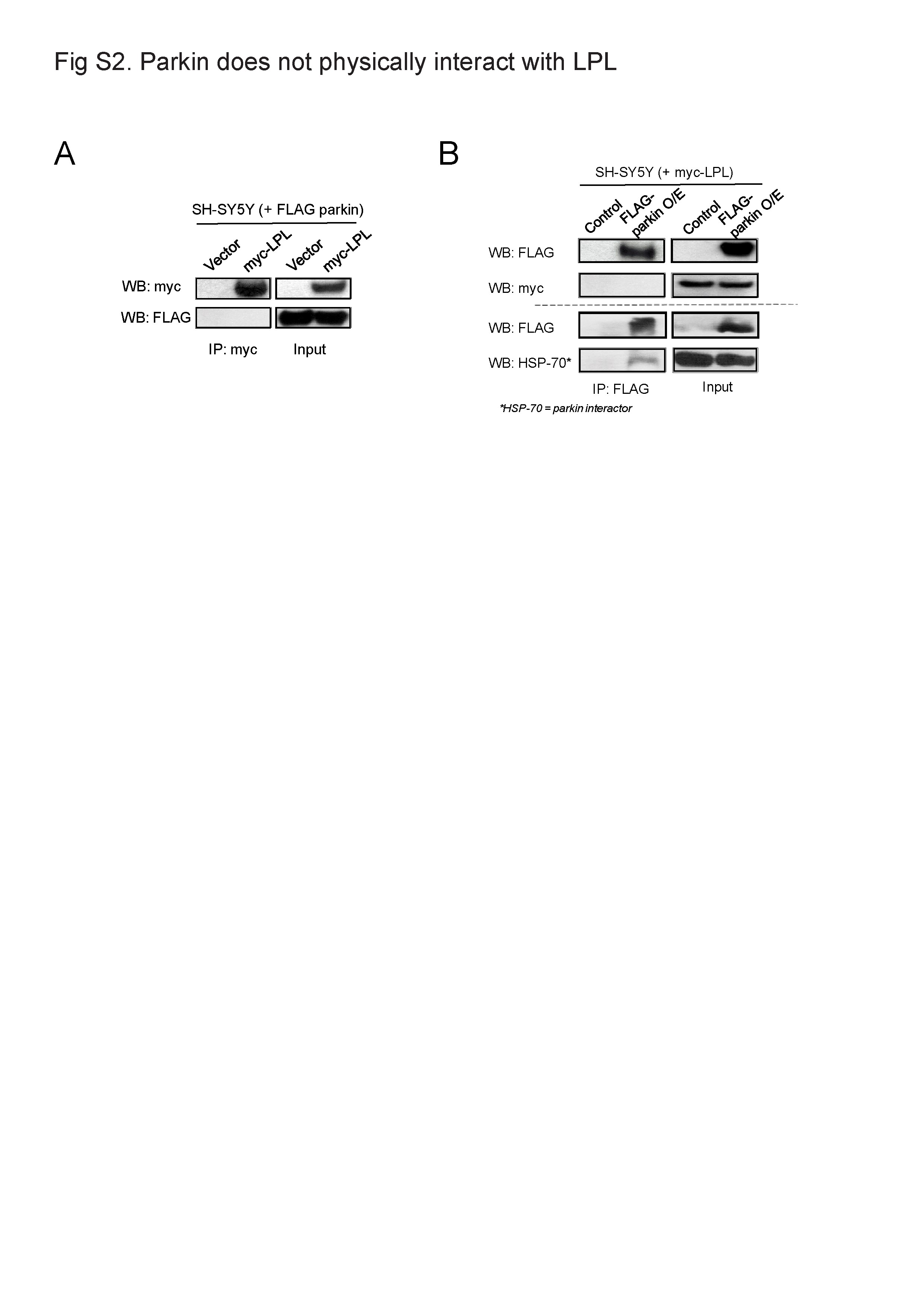

Supplement: FigS2_ddac297 [file figs2_ddac297.jpeg]

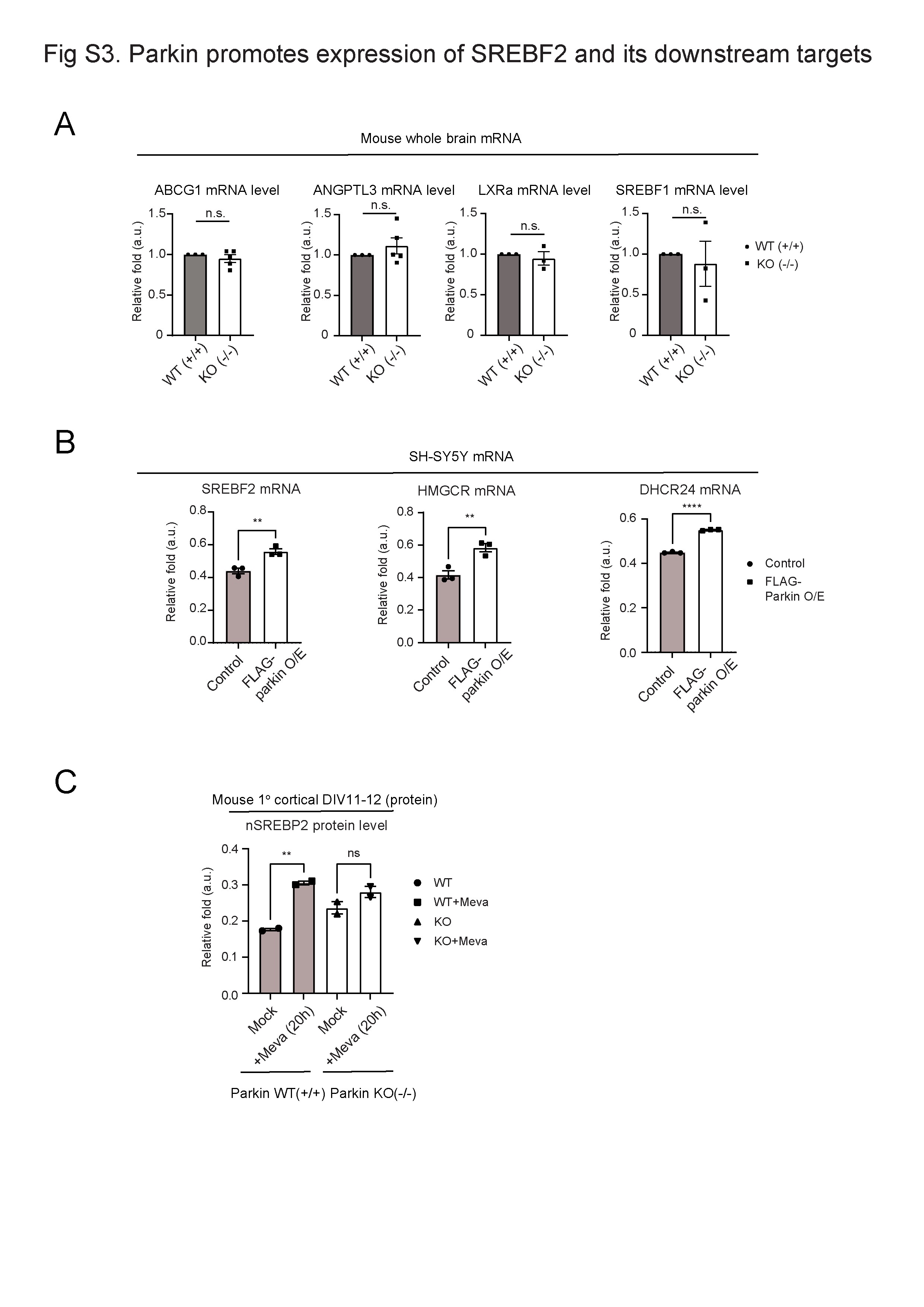

Supplement: FigS3_ddac297 [file figs3_ddac297.jpeg]

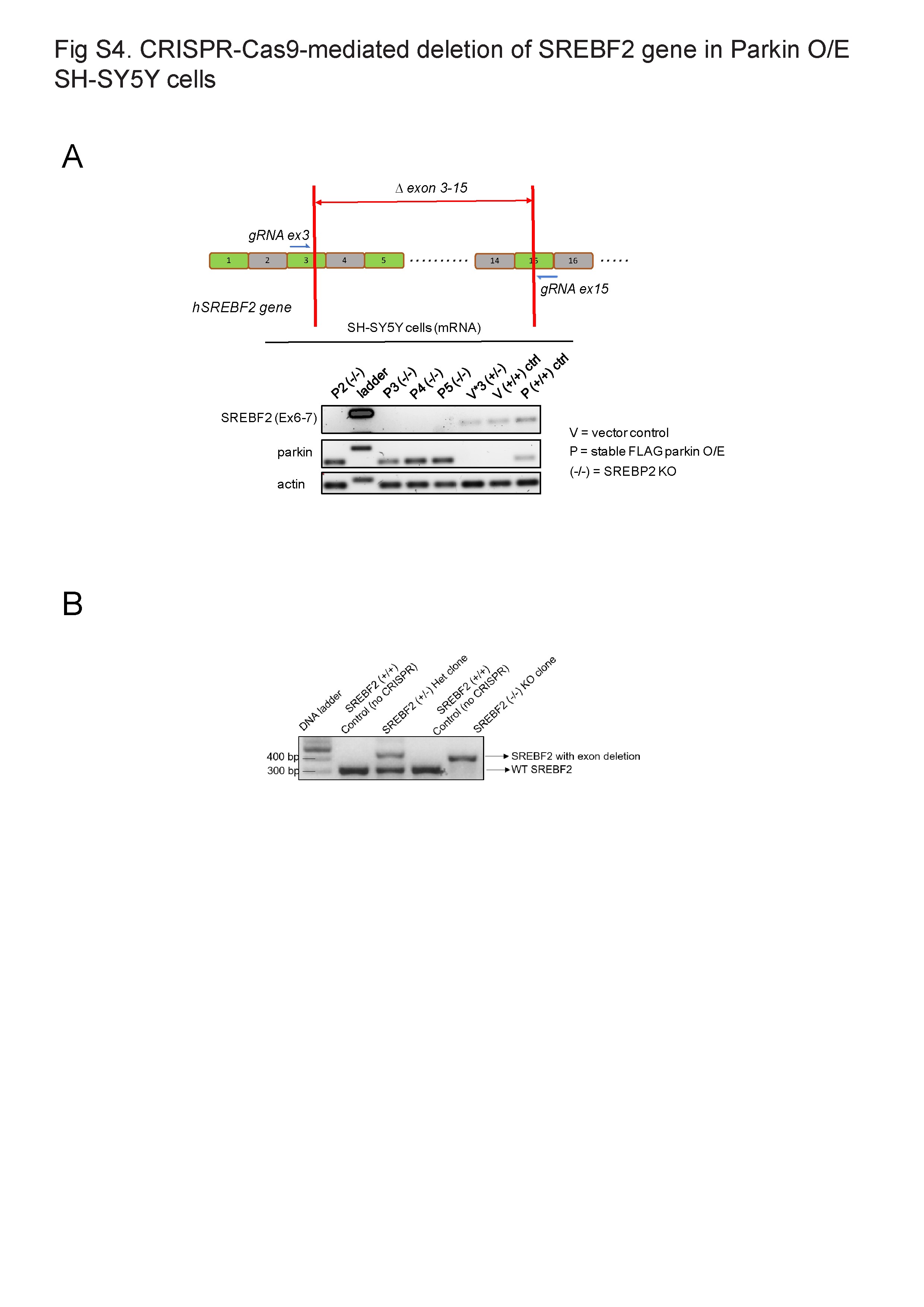

Supplement: FigS4_ddac297 [file figs4_ddac297.jpeg]

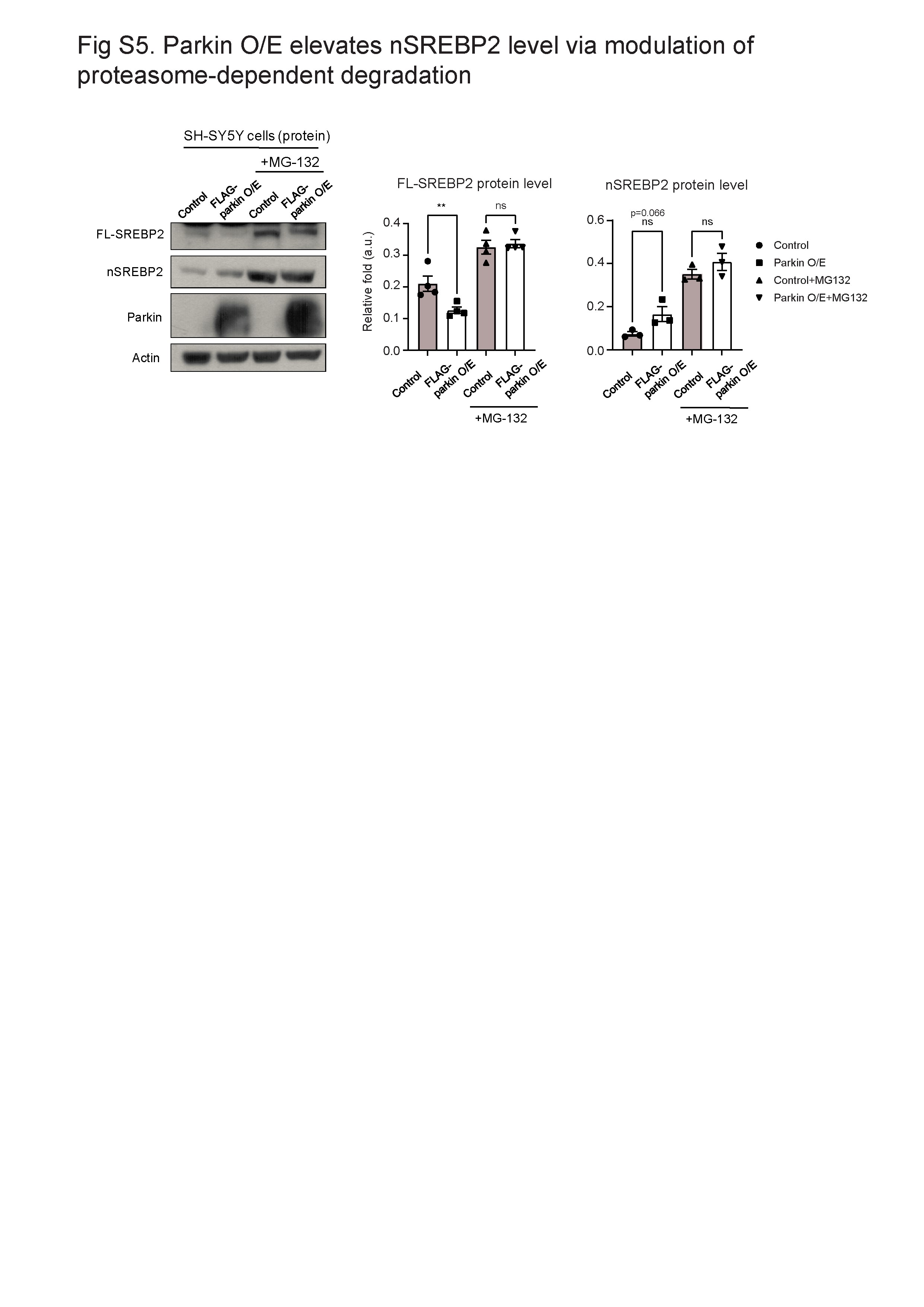

Supplement: FigS5_ddac297 [file figs5_ddac297.jpeg]

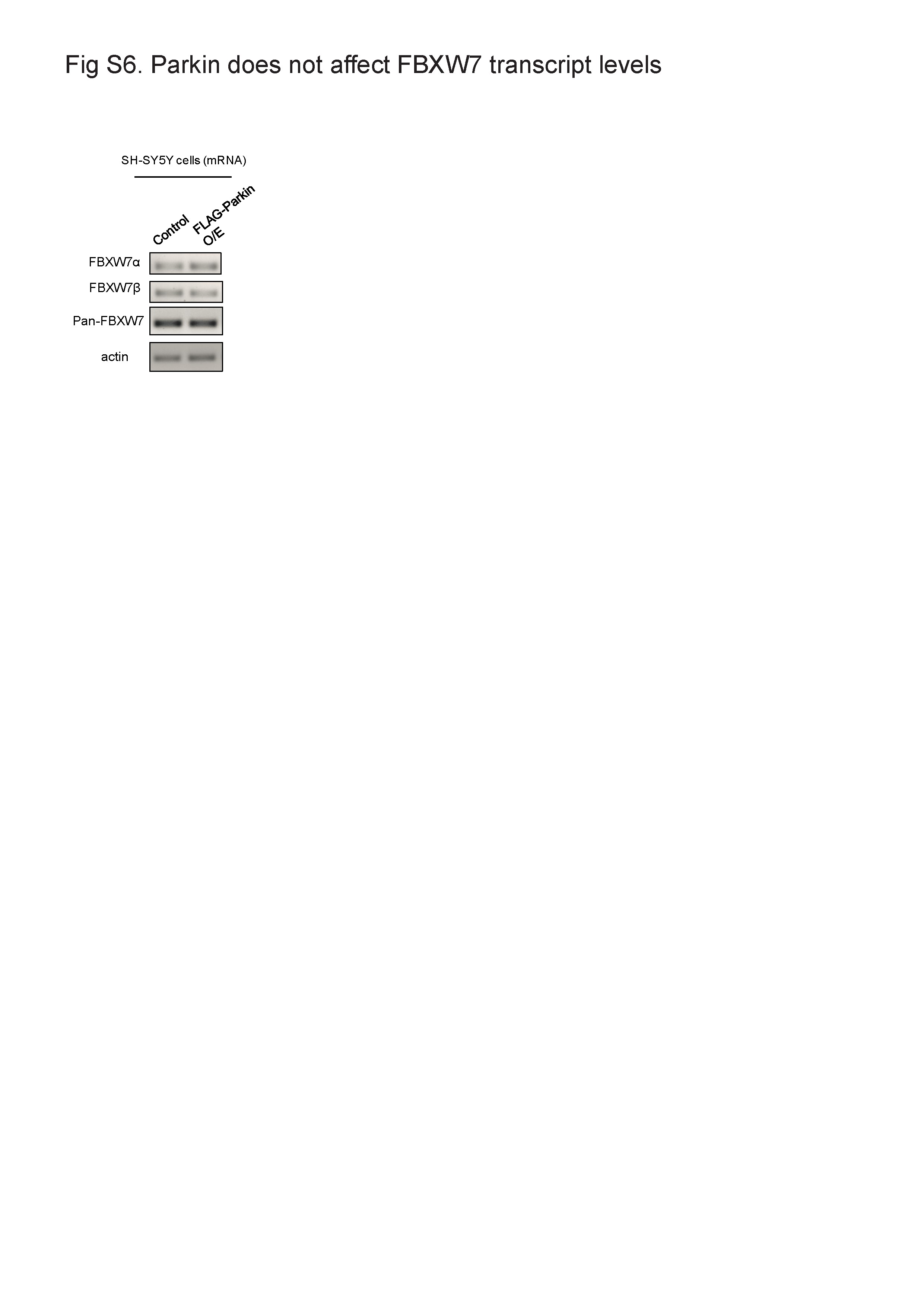

Supplement: FigS6_ddac297 [file figs6_ddac297.jpeg]

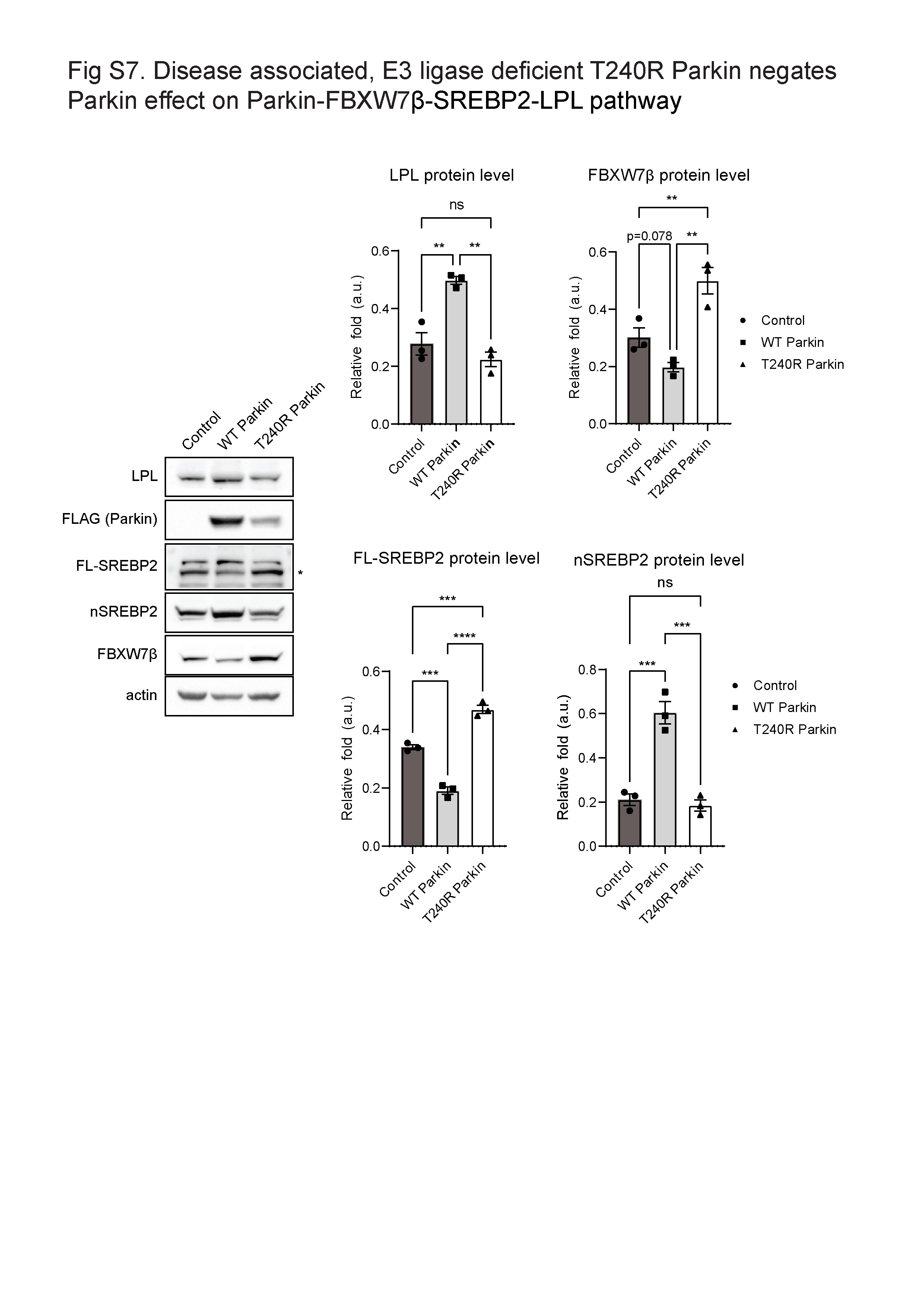

Supplement: FigS7_ddac297 [file figs7_ddac297.jpeg]

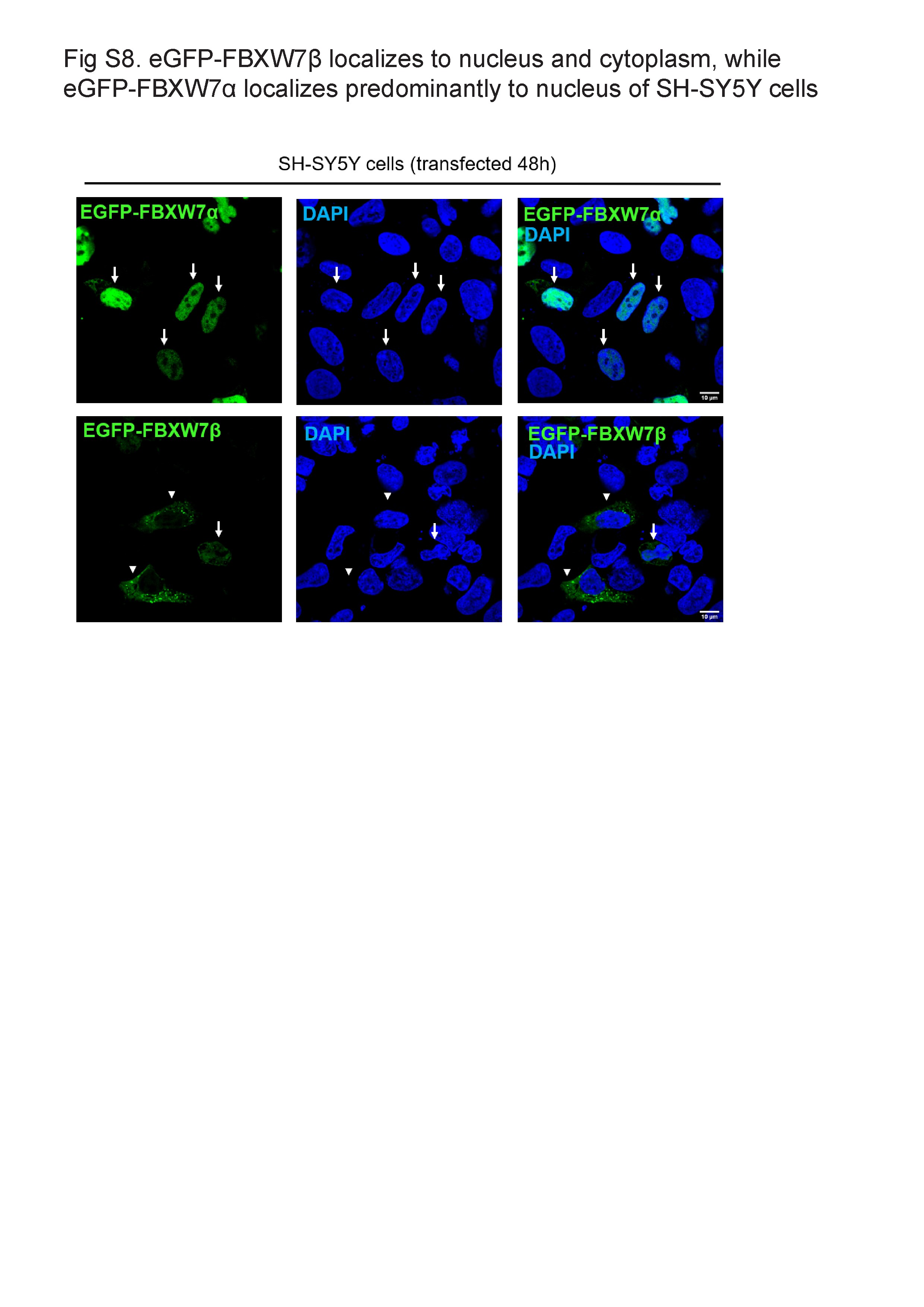

Supplement: FigS8_ddac297 [file figs8_ddac297.jpeg]

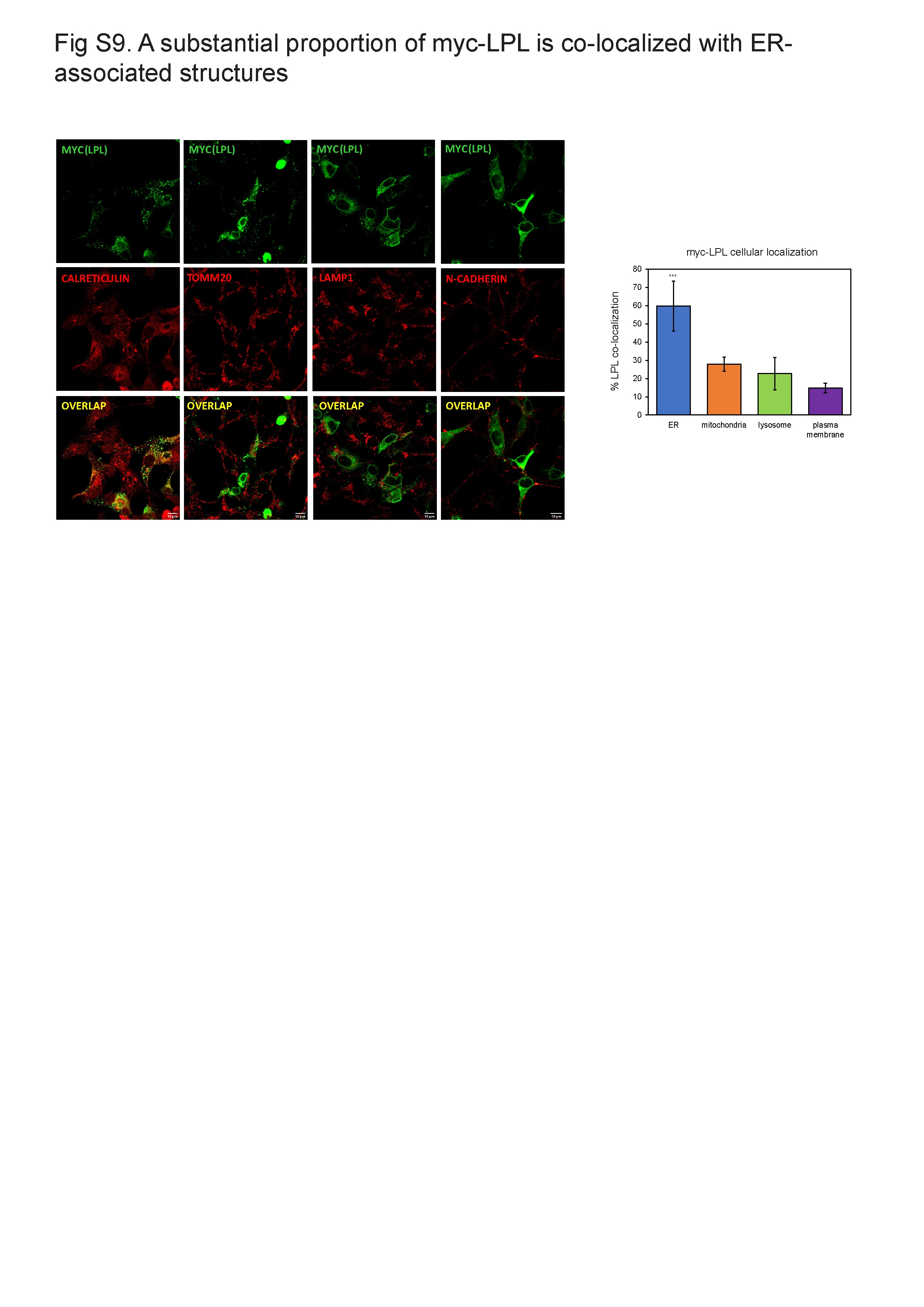

Supplement: FigS9_ddac297 [file figs9_ddac297.jpeg]

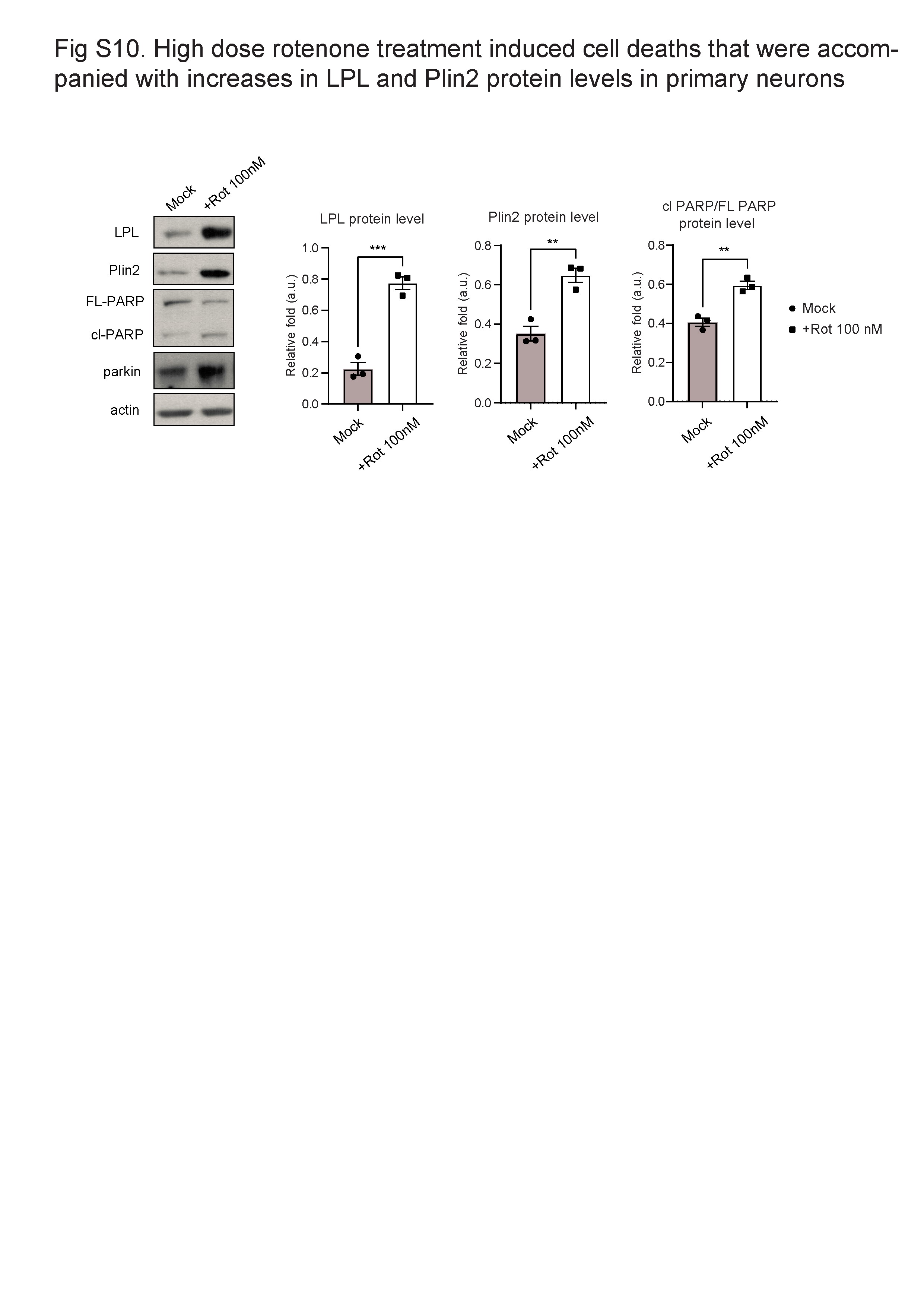

Supplement: FigS10_ddac297 [file figs10_ddac297.jpeg]

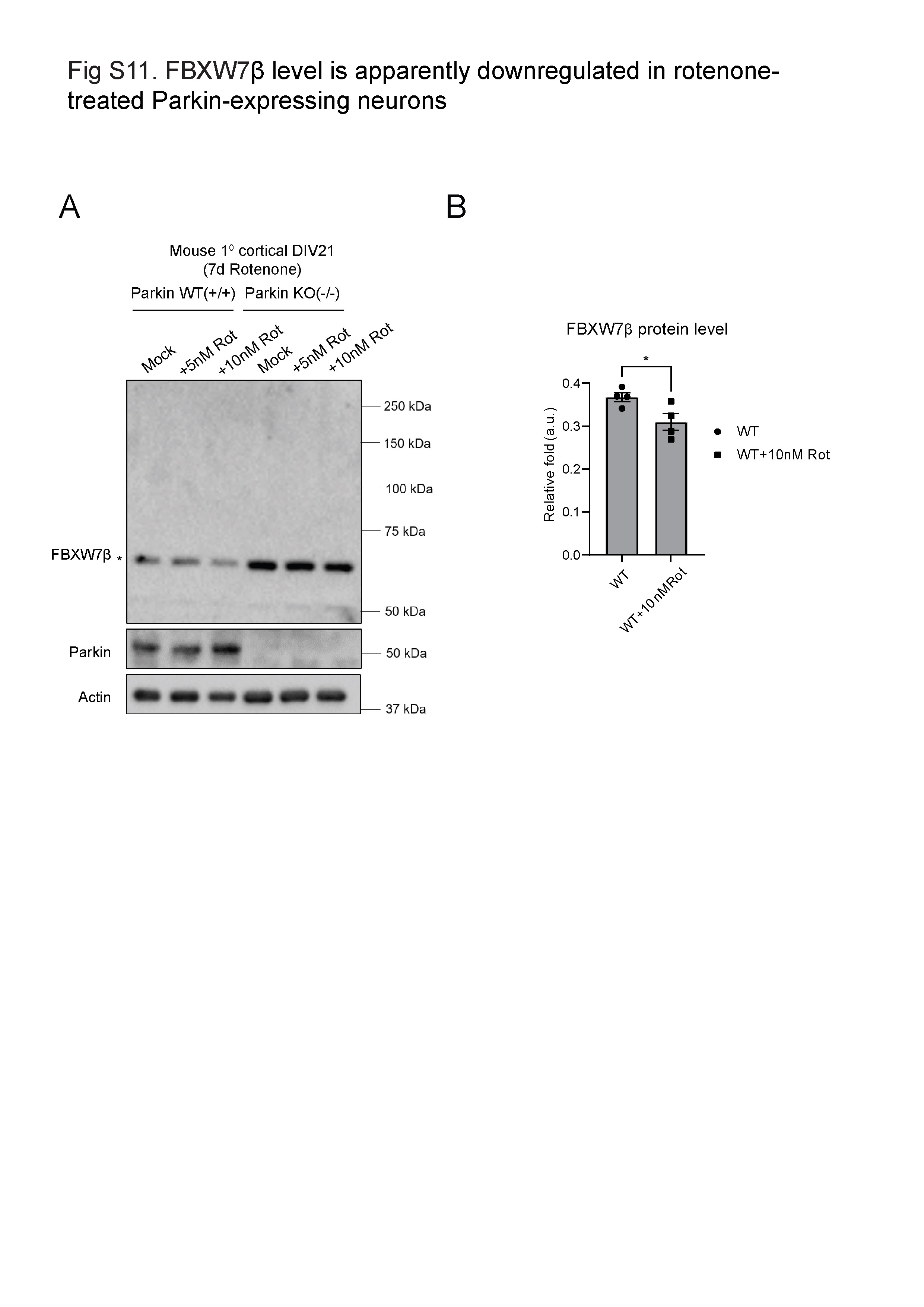

Supplement: FigS11_ddac297 [file figs11_ddac297.jpeg]

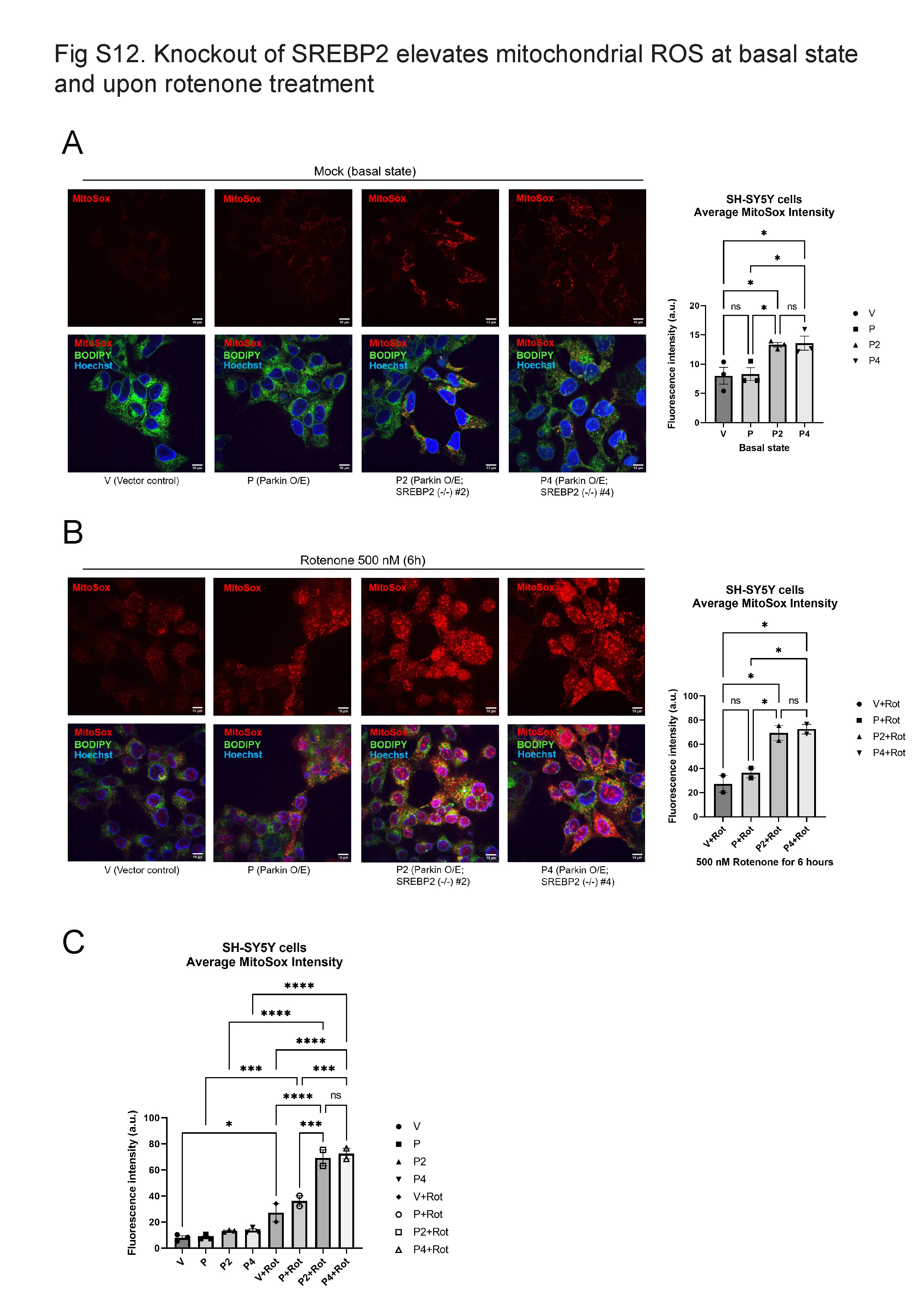

Supplement: FigS12_ddac297 [file figs12_ddac297.jpeg]

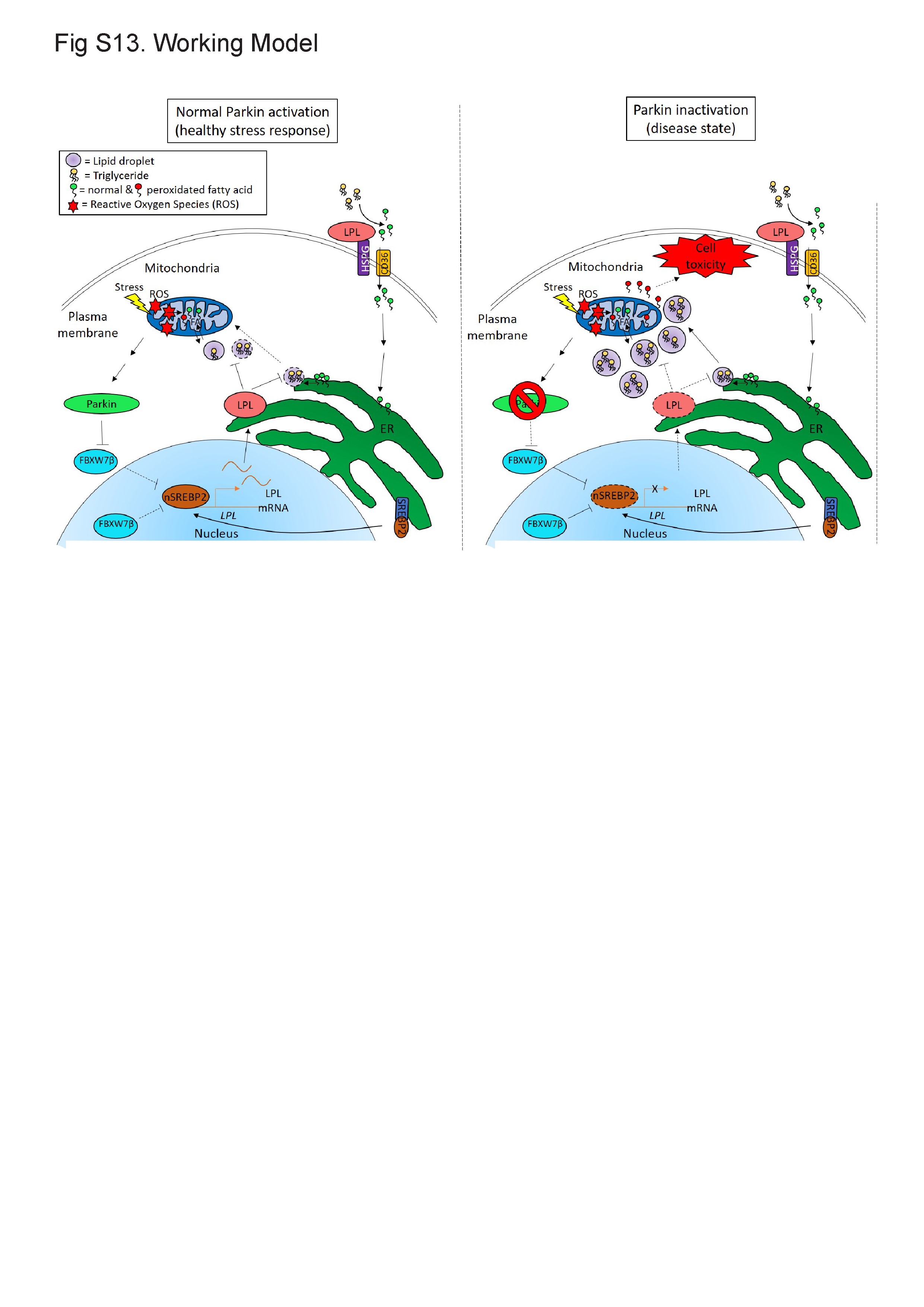

Supplement: FigS13_ddac297 [file figs13_ddac297.jpeg]

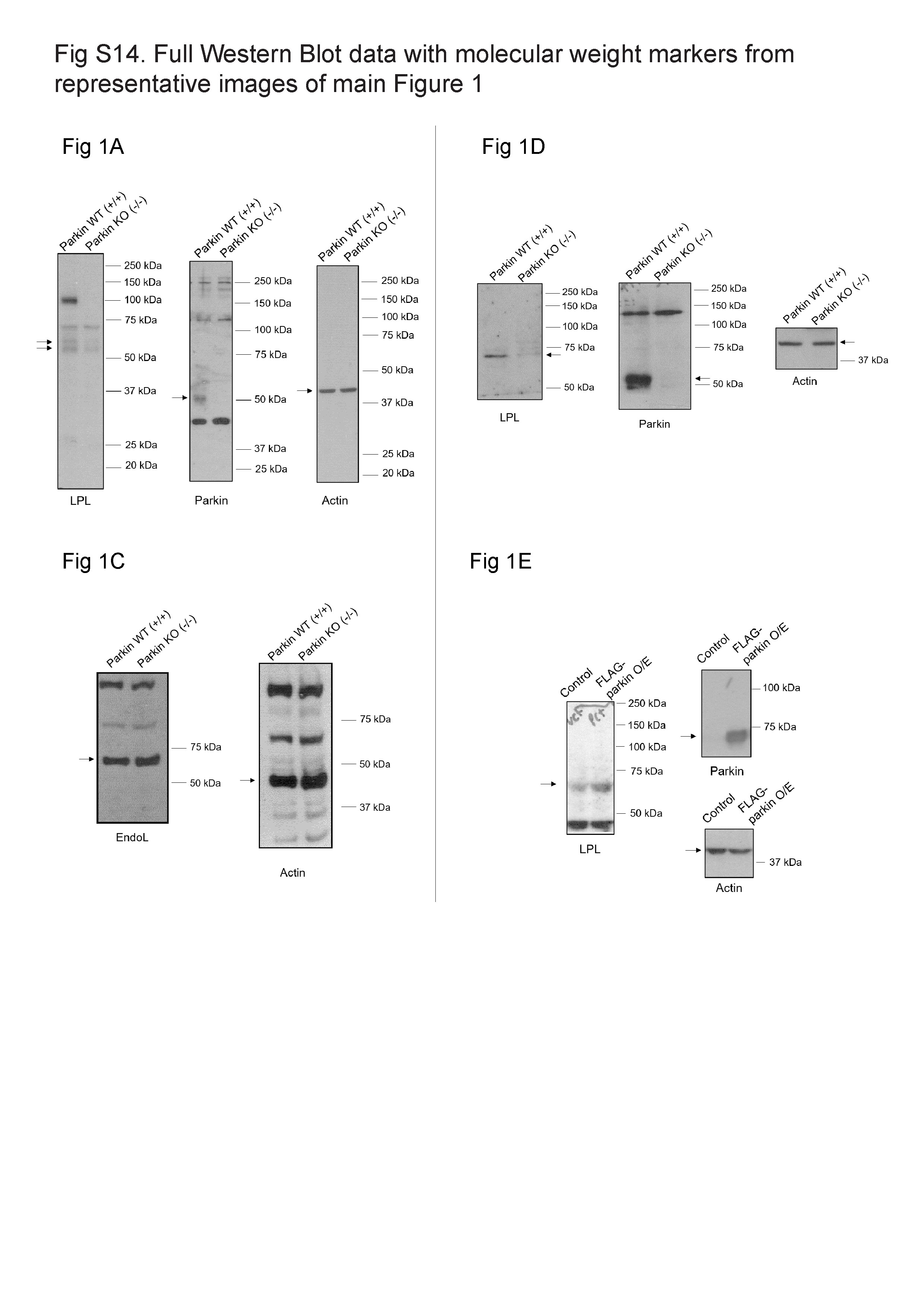

Supplement: FigS14_ddac297 [file figs14_ddac297.jpeg]

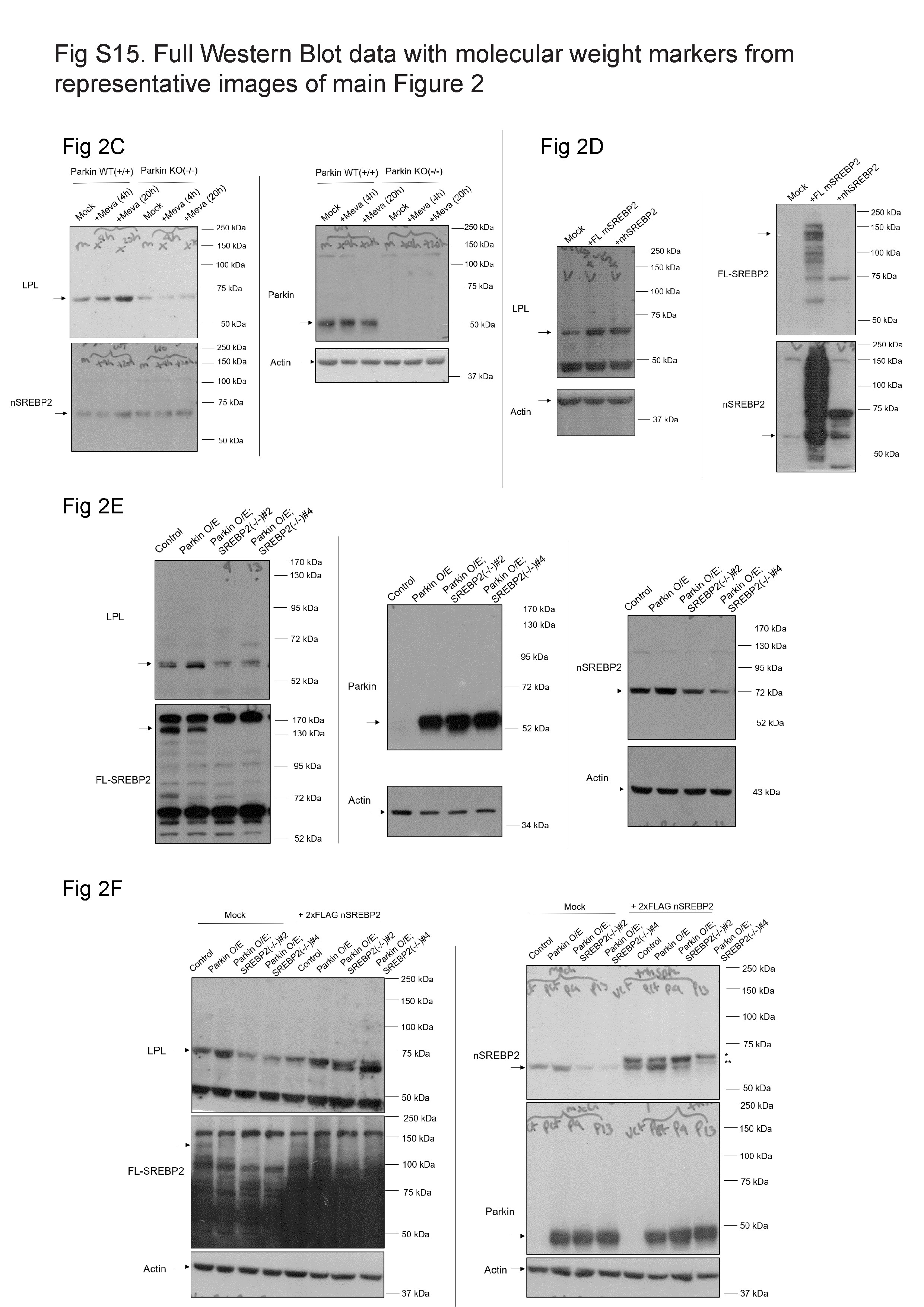

Supplement: FigS15_ddac297 [file figs15_ddac297.jpeg]

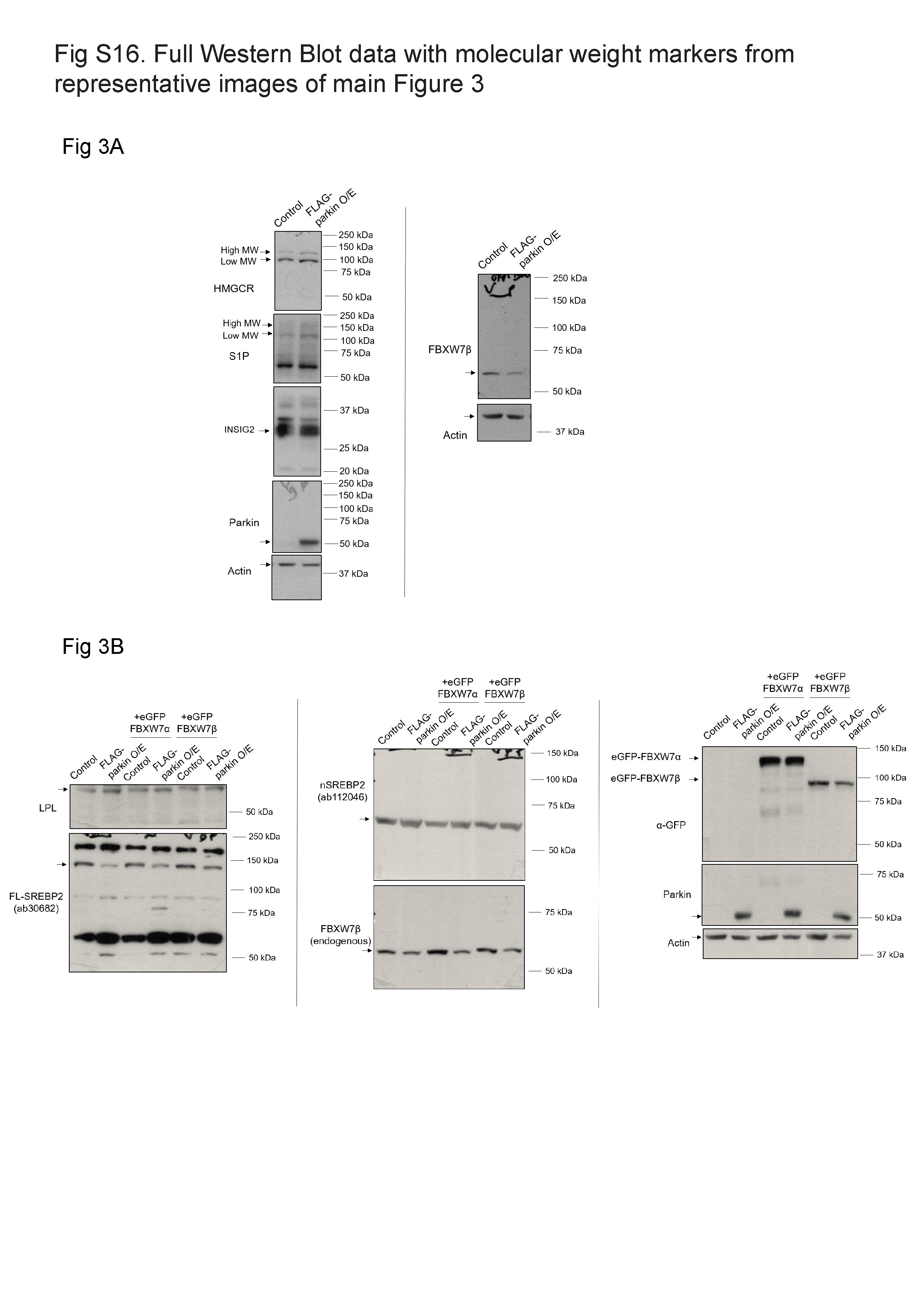

Supplement: FigS16_ddac297 [file figs16_ddac297.jpeg]

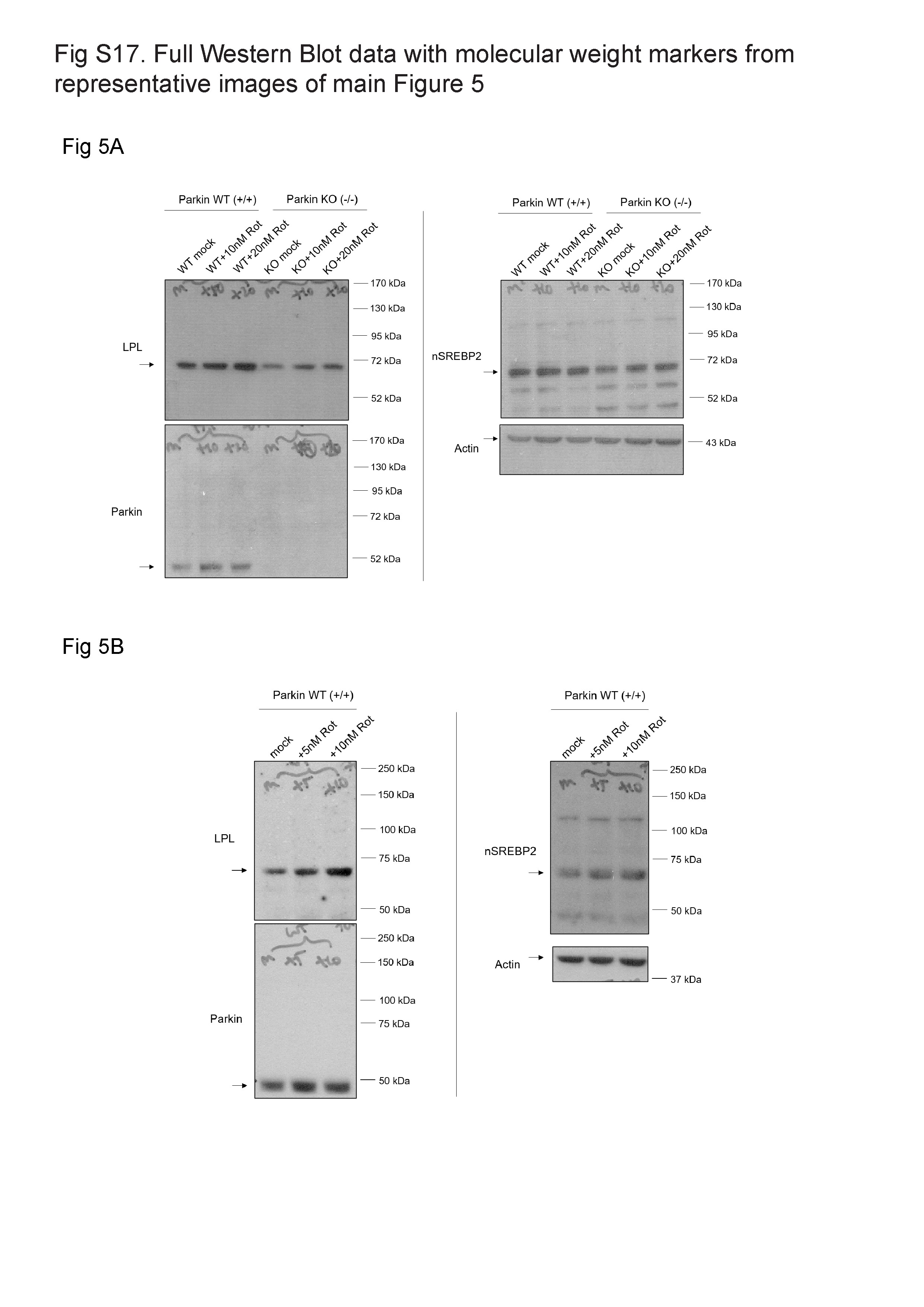

Supplement: FigS17_ddac297 [file figs17_ddac297.jpeg]
