# Supplementary material for: Parkin regulates neuronal lipid homeostasis through SREBP2-lipoprotein lipase pathway—implications for Parkinson’s disease
Source: Hum Mol Genet. 2022 Nov 26;32(9):1466–82. doi: 10.1093/hmg/ddac297 (PMC10117165; doi:10.1093/hmg/ddac297)
Supplement: Supplementary_legends_2nd_rev_HMG-2022-CE-00394_R1_ddac297 [file supplementary_legends_2nd_rev_hmg-2022-ce-00394_r1_ddac297.docx]

# **Supporting information**

**Fig. S1. Parkin does not affect CD36 expression level in the brain, but promotes LPL expression in other tissues. (A)** CD36 protein expression levels in whole brain lysates from adult Parkin (+/+) and Parkin (-/-) mice. **(B-D)** LPL protein expression levels (detected with LPL A.4 antibody) in **(B)** heart, **(C)** muscle, and **(D)** liver lysates from adult Parkin (+/+) and Parkin (-/-) mice. Actin serves as protein loading controls. (n = 3 biological replicates).

**Fig. S2. Parkin does not physically interact with LPL.** **(A)** Co-Immunoprecipitation (co-IP) of myc-LPL (with anti-myc antibody) followed by immunoblotting of FLAG-Parkin (with anti-FLAG antibody) from lysates of SH-SY5Y cell co-expressing FLAG-Parkin and myc-LPL or vector control. **(B)** Co-IP of FLAG-Parkin (with anti-FLAG antibody) followed by immunoblotting of either myc-LPL with anti-myc antibody (upper panel) or HSP-70 with anti-HSP-70 antibody (lower panel) from lysates of SH-SY5Y cell lysates co-expressing myc-LPL and FLAG-Parkin versus vector control. HSP-70 is a known interactor of Parkin and serves as a positive control for physical interaction.

**Fig. S3. Parkin promotes expression of SREBF2 and its downstream targets.** **(A)** Quantification of mRNA levels of *ABCG1*, *ANGPTL3*, *LXRa*, and *SREBF1* in Parkin (+/+) and Parkin (-/-) whole brain lysates from Fig. 2A. (n.s. = not statistically significant (t-test)). GAPDH mRNA level serves as loading controls **(B)** Quantification of mRNA levels of *SREBF2* and its downstream transcription targets, *HMCGR* and *DHCR24*, in lysates from SH-SY5Y cells stably overexpressing FLAG-Parkin or vector control from Fig. 2B (**p < 0.01; ****p< 0.0001 (t-test), n = 3 biological replicates). (*HMGCR* = 3-Hydroxy-3-Methylglutaryl-CoA Reductase; *DHCR24* = 24-Dehydrocholesterol Reductase). Actin mRNA level serves as loading controls. **(C)** Quantification of nSREBP2 protein levels as in Fig. 2C. nSREBP2 protein expression levels in Parkin (+/+) and Parkin (-/-) mouse primary cortical neurons DIV12-14 treated with the HMCGR inhibitor, Mevastatin (20 μM) for 20 hours compared to mock-treated cells (n.s. = not statistically significant; **p < 0.01 (ANOVA), n = 2 biological replicates).

**Fig. S4. CRISPR-Cas9-mediated deletion of SREBF2 gene in Parkin O/E SH-SY5Y cells.**

**(A)** Generation of SREBP2 (-/-) SH-SY5Y cells that stably overexpressing FLAG-Parkin via CRISPR method. Schematic diagram showing CRISPR-mediated deletion of exon 3-15 of SREBF2 gene (SREBF2 Δex3-15) using 2 gRNAs (upper panel). RT-PCR data showing loss of SREBF2 mRNA expression in different clones of SREBF2 Δex3-15 SH-SY5Y cells that stably overexpressing FLAG-Parkin (i.e., P2-P5 (-/-) cells) compared to control cells without SREBF2 deletion (lower panel). P2(-/-) and P4(-/-) refer to Parkin O/E; SREBP2(-/-) #2 and -#4 cell line respectively in the main figures. **(B)** Multiplex genomic DNA PCR results obtained by using 2 pairs of primers to identify heterozygous and homozygous Δex3-15 genomic deletion of SREBF2 locus in CRISPR-edited cell clones. One set of primers was designed to detect the presence of wild-type SREBF2 locus and yields ~300 bp PCR product. Another set of primers was designed to detect the presence Δex3-15 genomic deletion and yields ~400 bp PCR product.

**Fig. S5. Parkin O/E elevates nSREBP2 level via modulation of proteasome-dependent degradation**. SREBP2 protein expression levels in Parkin O/E SH-SY5Y cells compared to control cells under proteasomal inhibition by MG-132 (1 μM, 20h). (n.s. = not statistically significant; **p < 0.01 (ANOVA), n = 4 biological replicates for FL-SREBP2 & n = 3 biological replicates for nSREBP2).

**Fig. S6. Parkin does not affect FBXW7 transcript levels**. Measurement of mRNA levels of pan or individual FBXW7 isoforms in Parkin O/E versus control SH-SY5Y cells by RT-PCR. FBXW7γ mRNA expression was undetectable.

**Fig. S7. Disease associated, E3 ligase deficient T240R Parkin negates Parkin effect on Parkin-FBXW7β-SREBP2-LPL pathway**. Immunoblot data showing protein expression levels of LPL, FBXW7β, FL-SREBP2, and nSREBP2 in SH-SY5Y cell lines stably overexpressing either wild-type (WT) Parkin or T240R Parkin mutant compared to cells with vector controls (n.s. = not statistically significant; **p < 0.01; *** p < 0.001; ****p< 0.0001 (ANOVA), n=3 biological replicates). Actin serves as protein loading controls. * points to FL-SREBP2 protein band.

**Fig. S8. eGFP-FBXW7β localizes to nucleus and cytoplasm, while eGFP-FBXW7α localizes predominantly to nucleus of SH-SY5Y cells.** Representative fluorescence images of wild-type SH-SY5Y cells transfected with DNA constructs (Lipofectamine for 48 hours) that express either eGFP-FBXW7α or eGFP-FBXW7β. Arrows point to FBXW7 protein fraction that localizes to nucleus, while arrowheads point to FBXW7 protein fraction that localizes to cytoplasm. Cell nuclei were visualized with DAPI (blue). Scale bar = 10 µm

**Fig. S9. A substantial proportion of myc-LPL is co-localized with ER-associated structures. (Left panel)** Series of confocal fluorescence images of myc-LPL O/E SH-SY5Y cells co-immunostained with LPL A.4 antibody and organelle markers, such as Calreticulin (ER), Tomm20 (mitochondria), LAMP1 (lysosome), and N-Cadherin (plasma membrane). Scale bar = 10 µm **(Right panel)** Quantification of co-localization area between myc-LPL and organelle markers. The proportion of co-localized area is measured by the percentage of co-localized LPL area over total LPL^+^ area. (***p < 0.001 (ANOVA) for Calreticulin samples versus each of other organelle markers; n = 65 cells (Calreticulin), 27 cells (Tomm20), 20 cells (LAMP1), and 35 cells (N-Cadherin)).

**Fig. S10. High dose rotenone treatment induced cell deaths that were accompanied with increases in LPL and Plin2 protein levels in primary neurons.** Immunoblotting data for LPL (detected with LPL A.4 antibody), Plin2, full-length (FL) PARP, cleaved (Cl) PARP, and Parkin in WT mouse primary cortical neurons DIV11 treated with 100nM rotenone for 24 hours. Actin serves as protein loading controls. (**p < 0.01; ***p < 0.001 (t-test), n = 3 biological replicates).

**Fig. S11. FBXW7β level is apparently downregulated in rotenone-treated Parkin-expressing neurons. (A)** Representative Western blot data showing FBXW7β protein expression level in Parkin WT (+/+) and Parkin KO (-/-) mouse primary cortical neurons DIV21 treated with 0, 5, or 10 nM rotenone for 7 days. **(B)** Western blot quantification of FBXW7β protein level in Parkin WT neurons DIV16-21 treated with 10 nM rotenone for 7 days compared to untreated controls (*p < 0.05 (t-test), n = 4 biological replicates).

**Fig. S12. Knockout of SREBP2 elevates mitochondrial ROS at basal state and upon rotenone treatment. (A-B)** Representative live confocal fluorescence images of FLAG Parkin O/E;SREBP2(-/-) SH-SY5Y cells together with the Parkin O/E parental line and vector control grown in either (A) galactose supplemented media (basal state) only or (B) galactose supplemented media with 500 nM rotenone for 6 hours . Mitochondrial ROS level was measured via MitoSOX (red) fluorescence intensity and the quantifications are presented on the right panel. (n.s. = not statistically significant; *p < 0.05 (ANOVA): Control (572 cells, 29 fields), Parkin O/E (723 cells, 29 fields), P2 (804 cells, 30 fields), P4 (584 cells, 29 fields), Control+Rot (677 cells, 22 fields), Parkin O/E+Rot (528 cells, 21 fields), P2+Rot (711 cells, 18 fields), P4+Rot (563 cells, 20 fields), n = 3 biological replicates for basal state samples and 2 biological replicates for rotenone-treated samples). BODIPY 493/503 (green) and Hoechst staining was used to identify individual cell body and nucleus, respectively. Scale bar = 10 µm. **(C)** Side-by-side comparison of average MitoSox intensity for samples in (A-B) showing marked elevation of MitoSOX signal in all tested conditions upon rotenone treatment (n.s. = not statistically significant; *p < 0.05; ***p < 0.001; ****p < 0.0001 (ANOVA)).

**Fig. S13. Working model of Parkin-SREBP2-LPL pathway.**  (**Left panel**) In normal physiological condition, extracellular LPL binds to its plasma membrane receptor (e.g., HSPG) to promote hydrolysis of circulating triglycerides (TGs) to free fatty acids (FAs), which are then taken up intracellularly by CD36 for basal energy maintenance of the cell. Neuronal exposure to PD-linked mitochondria stressors (e.g., rotenone) triggers oxidative stress and initiates the activation of Parkin and its associated downstream neuroprotective pathways. Parkin then promotes the degradation of FBXW7β, which subsequently reduces nuclear SREBP2 (nSREBP2) basal degradation. Elevated level of nSREBP2 then promotes general upregulation of LPL allowing more LPL to be localized in endoplasmic reticulum (ER). ER-localized LPL then modulates lipid droplet (LD) synthesis from ER by controlling the amount of ER TGs via its lipid hydrolysis activity. This Parkin-dependent upregulation of LPL ultimately prevents aberrant build-up of intracellular LDs due to compromised mitochondria lipid regulatory function that can be detrimental to the neuronal cells. **(Right panel)** In disease state with Parkin loss of function, neuronal cells with mitochondria dysfunctions are unable to upregulate ER-localized LPL via Parkin-SREBP2-LPL pathway. This in turn triggers excessive LD accumulation and exacerbates further the ineffective FA utilization by mitochondria. Progressive elevation of FAs that are prone to be peroxidated by ROS in turn will trigger neurotoxic cascade that leads to neurodegeneration. This molecular mechanism may be implicated in dopaminergic neurodegeneration seen in PD brains.­­­­­­­­­

**Fig. S14. Full Western Blot data with molecular weight markers from representative images of main Figure 1.** Arrows denote the protein bands of interest. Molecular weight markers are presented on right side of each blot in kilodalton (kDa).

**Fig. S15. Full Western Blot data with molecular weight markers from representative images of main Figure 2.** Arrows denote the protein bands of interest. Molecular weight markers are presented on right side of each blot in kilodalton (kDa).

**Fig. S16. Full Western Blot data with molecular weight markers from representative images of main Figure 3.** Arrows denote the protein bands of interest. Molecular weight markers are presented on right side of each blot in kilodalton (kDa).

**Fig. S17. Full Western Blot data with molecular weight markers from representative images of main Figure 5.** Arrows denote the protein bands of interest. Molecular weight markers are presented on right side of each blot in kilodalton (kDa).
